# ATXN2-CAG42 Sequesters PABPC1 into Insolubility and Induces FBXW8 in Cerebellum of Old Ataxic Knock-In Mice

**DOI:** 10.1371/journal.pgen.1002920

**Published:** 2012-08-30

**Authors:** Ewa Damrath, Melanie V. Heck, Suzana Gispert, Mekhman Azizov, Joachim Nowock, Carola Seifried, Udo Rüb, Michael Walter, Georg Auburger

**Affiliations:** 1 Experimental Neurology, Department of Neurology, Goethe University Medical School, Frankfurt am Main, Germany; 2 Department of Clinical Neuroanatomy, Dr. Senckenbergisches Chronomedizinisches Institut, Goethe University Medical School, Frankfurt am Main, Germany; 3 Institute of Medical Genetics, Eberhard Karls University, Tübingen, Germany; University of Minnesota, United States of America

## Abstract

Spinocerebellar Ataxia Type 2 (SCA2) is caused by expansion of a polyglutamine encoding triplet repeat in the human *ATXN2* gene beyond (CAG)_31_. This is thought to mediate toxic gain-of-function by protein aggregation and to affect RNA processing, resulting in degenerative processes affecting preferentially cerebellar neurons. As a faithful animal model, we generated a knock-in mouse replacing the single CAG of murine *Atxn2* with CAG42, a frequent patient genotype. This expansion size was inherited stably. The mice showed phenotypes with reduced weight and later motor incoordination. Although brain *Atxn2* mRNA became elevated, soluble ATXN2 protein levels diminished over time, which might explain partial loss-of-function effects. Deficits in soluble ATXN2 protein correlated with the appearance of insoluble ATXN2, a progressive feature in cerebellum possibly reflecting toxic gains-of-function. Since *in vitro* ATXN2 overexpression was known to reduce levels of its protein interactor PABPC1, we studied expansion effects on PABPC1. In cortex, PABPC1 transcript and soluble and insoluble protein levels were increased. In the more vulnerable cerebellum, the progressive insolubility of PABPC1 was accompanied by decreased soluble protein levels, with PABPC1 mRNA showing no compensatory increase. The sequestration of PABPC1 into insolubility by ATXN2 function gains was validated in human cell culture. To understand consequences on mRNA processing, transcriptome profiles at medium and old age in three different tissues were studied and demonstrated a selective induction of *Fbxw8* in the old cerebellum. *Fbxw8* is encoded next to the *Atxn2* locus and was shown *in vitro* to decrease the level of expanded insoluble ATXN2 protein. In conclusion, our data support the concept that expanded ATXN2 undergoes progressive insolubility and affects PABPC1 by a toxic gain-of-function mechanism with tissue-specific effects, which may be partially alleviated by the induction of FBXW8.

## Author Summary

Frequent age-associated neurodegenerative disorders like Alzheimer's, Parkinson's, and Lou Gehrig's disease are being elucidated molecularly by studying rare heritable variants. Various hereditary neurodegenerative disorders are caused by polyglutamine expansions in different proteins. In spite of this common pathogenesis and the pathological aggregation of most affected proteins, investigators were puzzled that the pattern of affected neuron population varies and that molecular mechanisms seem different between such disorders. The polyglutamine expansions in the Ataxin-2 (ATXN2) protein are exceptional in view of the lack of aggregate clumps in nuclei of affected Purkinje neurons and well documented alterations of RNA processing in the resulting disorders SCA2 and ALS. Here, as a faithful disease model and to overcome the unavailability of autopsied patient brain tissues, we generated and characterized an ATXN2-CAG42-knock-in mouse mutant. Our data show that the unspecific, chronically present mutation leads to progressive insolubility and to reduced soluble levels of the disease protein and of an interactor protein, which modulates RNA processing. Compensatory efforts are particularly weak in vulnerable tissue. They appear to include the increased degradation of the toxic disease protein by FBXW8. Thus the link between protein and RNA pathology becomes clear, and crucial molecular targets for preventive therapy are identified.

## Introduction

Spinocerebellar Ataxia Type 2 (SCA2) is one of 9 currently known inherited neurodegenerative diseases (e.g. Huntington's disease, SCA1, SCA3, SBMA) that are caused by an expanded CAG trinucleotide repeat within the coding region of the disease gene, which expands its size from generation to generation and is translated to a polyglutamine (polyQ) domain [1]–[4]. More than 90% of the human population carry a repeat size of 22–23 triplets in the Ataxin-2 (*ATXN2*) gene [3], while alleles between 27 and 33 are considered intermediate size expansions and were recently shown to result in a higher risk for related neurodegenerative diseases such as ALS and Parkinsonism [5]–[7]. Individuals having a polyQ-repeat of 32 CAGs or more may develop SCA2 [8], with larger repeat sizes resulting in a more severe disease course and earlier manifestation. Clinical disease onset occurs usually in late adult life [9]–[11].

The clinical manifestations of all Spinocerebellar Ataxias are similar, making the molecular genotyping indispensable to establish diagnosis [8]. Increased appetite with subsequent loss of subcutaneous fat and of weight are peripheral tissue features in other polyQ neurodegenerative diseases such as Huntington's disease [12], [13] and are also common in SCA2 patients [8], [14]. The primary characteristic manifestation in SCA2 patients reflects cerebellar incoordination signs like gait/stance/limb ataxia, dysarthria, dysmetria, adiadochokinesia, action tremor and hypotonia. Besides, the thalamus, brainstem, cranial nerves, spinal cord and muscles are affected early on, leading to a relatively characteristic manifestation with reduced saccade velocity, altered sleep, reduced deep tendon reflexes and cramps [15]–[23]. Early involvement of the midbrain can also lead to a manifestation as Parkinsonism [24], [25]. Degeneration of the basal ganglia and the precerebellar nuclei is also present, while the affection of the cerebral cortex occurs relatively late [15], [26]. Parkinsonism is also frequent among the few patients with SCA2 homozygosity, where generally the same vulnerability pattern was observed, but relatively early affection of the retina, the oculomotor, reticulotegmental, facial, lateral vestibular, raphe interpositus nuclei in the brainstem and some pyramidal cells in the primary motor cortex was reported [27]–[29]. Therapy is mostly palliative, but a surprising effect of deep brain stimulation on tremor was reported in one case [30]. In the end stage of disease, patients die mostly from respiratory failure [8].

Microscopic SCA2 findings in the cerebellum include early loss of the large Purkinje neurons, preceded by a reduction in their dendritic arborisation and in the thickness of the molecular layer where the synaptic contacts and neuronal processes are located [15]. While pathological intranuclear inclusion bodies (NIIs) containing polyQ aggregates in SCA2 are detectable only in a minority of brainstem neurons and never in cerebellar Purkinje neurons, cytoplasmic aggregates of ATXN2 were reported in SCA2 patient brains and in a mouse mutant with Purkinje-cell specific maximal transgenic overexpression of ATXN2 [31]–[33], raising the question to what extent the formation of inclusion bodies containing the insoluble disease protein is the driving force in the neurodegenerative process of SCA2.

The expanded polyglutamine domain in other disease proteins is thought to mediate pathogenesis mainly by a toxic gain-of-function mechanism, in particular through aggregation of the mutant protein [34]. Partial loss-of-function effects have also been noted for polyQ disease proteins where the physiological function can already be quantified [35]–[37]. The few facts known about the function of ATXN2 suggest that it exerts its neurodegenerative effects as part of protein-RNA complexes [5]. ATXN2 is involved to some extent in trophic signalling [38]. Sequence analysis of ATXN2 revealed a PAM2 motif that mediates direct binding with the poly(A)-binding protein PABPC1 [39], [40] and two Lsm motifs that may be involved in RNA processing. In the mammalian cell line HEK293, the reduced ATXN2 levels lead to increased endogenous PABPC1 levels and vice versa [41]. ATXN2 protein associates with polyribosomes [40] and is mainly localized at the rough endoplasmic reticulum [42]. Furthermore, ATXN2 was found to be part of and necessary for the formation of stress granules (SGs), distinct foci within the cytoplasm, where untranslated mRNAs are translationally inhibited during conditions of cell stress [39], [41]. The expression of ATXN2 in the cerebellum is prominent in Purkinje neurons that comprise a small minority of all cerebellar cells, but are conspicuous due to their large cytoplasm and dendritic trees, and their exceptional content of ribosomal machinery easily visualized in Nissl stains. In cortex the expression of ATXN2 is widespread through neuron populations of several layers (compare Allen brain atlas and reference [43]).

As a faithful animal model of SCA2, we now generated the first ATXN2 polyQ knock-in mouse. While mice usually have 1 CAG at the site of the human ATXN2 triplet repeat, the knock-in mice were designed to carry 42 CAGs in this locus. Knock-in mice have three distinct advantages over available models [44]. Firstly, the preferential vulnerability of specific tissues can be investigated in contrast to models employing heterologous promoters. For this aim we chose to compare the cerebellum as an early, severely affected tissue versus cerebral cortex where the neurons are lost only at late stages and where cognitive signs originate only in few patients. Secondly, in knock-in mice the contribution of partial loss-of-function versus toxic gain-of-function effects can be studied, in contrast to transgenic overexpressing animals or transfected cells that model SCA2 through elevated dosage of ATXN2 DNA, mRNA and protein. For this purpose, we performed a careful evaluation of mutants from different litters in comparison to their wild-type littermates, assessing systematically the endogenous transcript levels, soluble protein and insoluble protein levels as driven by the endogenous promoter. Thirdly, knock-in mice have the advantage over knock-in cells that disease progression and mutation effects can be studied over years. Towards this goal, we decided to perform longitudinal observation from early adult life (age 6 weeks) to multimorbid senescence (age 21 months) and to document the progressive effects of the ATXN2 expansion on PABPC1. We chose PABPC1 as a direct protein interactor of ATXN2, which relocalizes together with ATXN2 under cell stress from the rER to SGs, is controlled in its protein level by the physiological function of ATXN2, and has a well studied cellular role in RNA processing. The data observed indicate selective and progressive pathology of cerebellar tissue regarding ATXN2 insolubility, PABPC1 deficiency, FBXW8 upregulation and locomotor impairment.

## Results

### The Repeat Size of *Atxn2*-CAG42-Knock-In Mice Is Stably Transmitted

**Figure 1:**
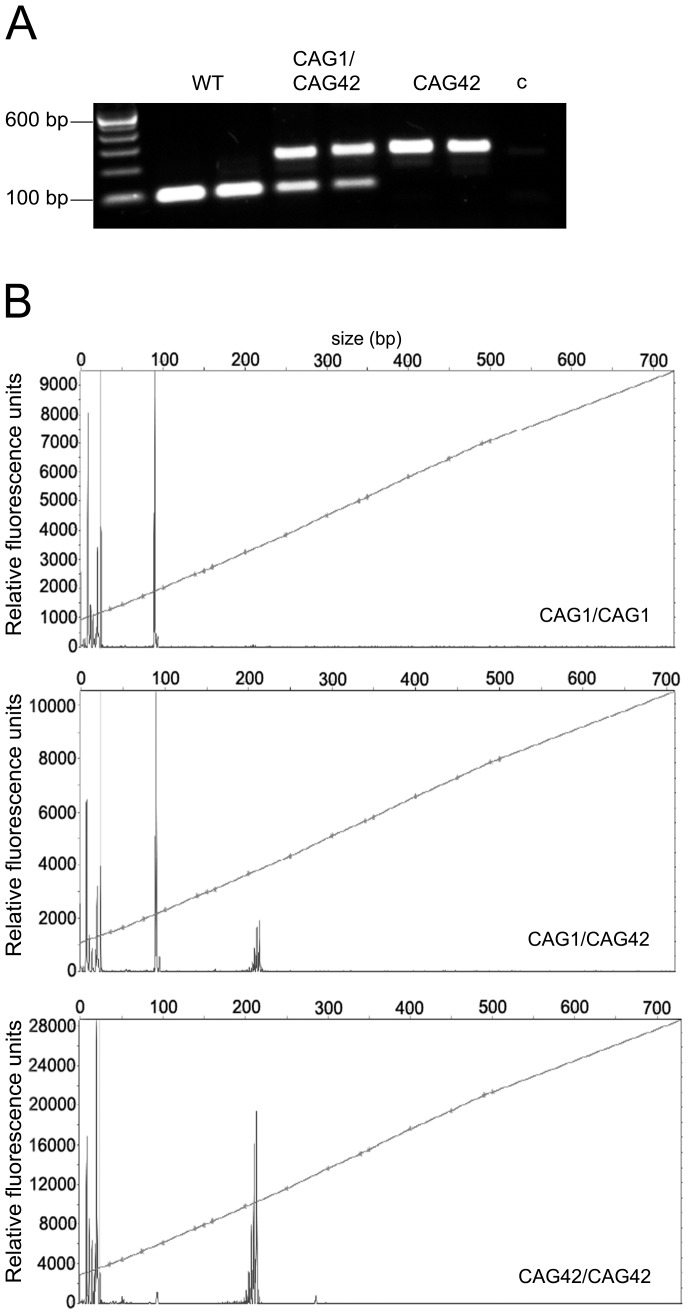
The paternal and maternal transmission of the 42 CAG repeat is stable. (A) A CAG-repeat flanking PCR with one FAM-labelled primer was performed. As expected, wild-type (WT) animals showed one product at 94 bp, whereas heterozygotes (CAG1/CAG42) showed an additional larger product. In homozygous mutant mice (CAG42) only the larger product was detected. c: negative control. (B) The exact PCR product size was confirmed by fragment length analysis. The peak in the WT sample represents the 94 bp product, while the 217 bp peak appeared in CAG42 animals. Both peaks were detectable in CAG1/CAG42 mice.

We generated a mouse line that expresses expanded *Atxn2* under the control of the endogenous murine *Atxn2* promoter (Figures S1 and S2, Table S1). To confirm the successful homologous recombination, a PCR with CAG-repeat flanking primers was performed on DNA tail biopsies (Figure 1A), the product sequence verified and the introduction of the CAG42-repeat confirmed. To test how the repeat is transmitted in the mice over successive generations, the PCR-products from mice across nine generations from WT, heterozygous (CAG1/CAG42) and homozygous (CAG42) animals were subjected to fragment analysis and their size was determined (Figure 1B). All products had the exact same length. These data indicate that the knock-in mice have a repeat of 42 CAGs that is stably transmitted.

### CAG42 Mice Show Reduced Weight and Change from Excellent Motor Coordination at Young Age to a Deficit at Old Age

**Figure 2:**
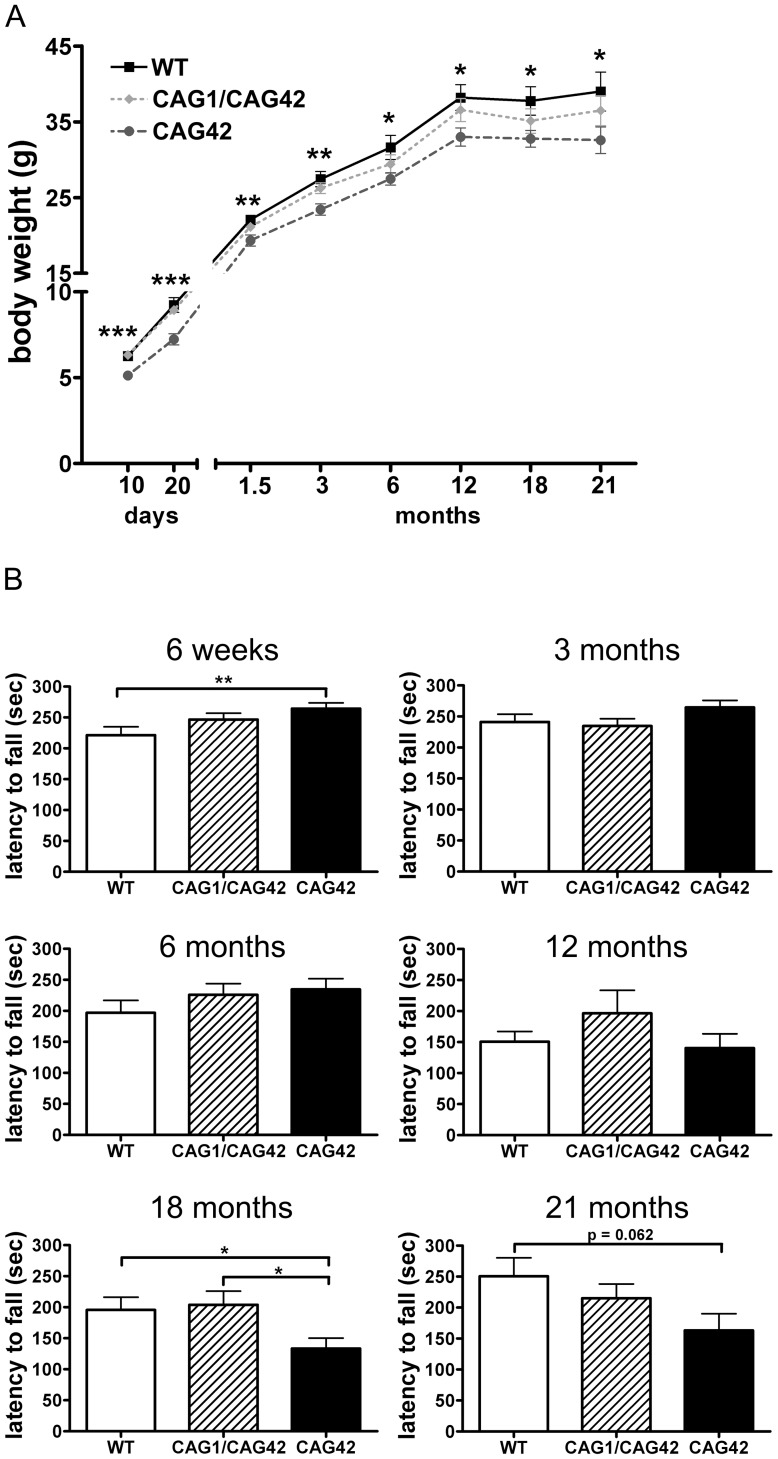
A constant weight reduction and a late-onset deficit in a cerebellar test paradigm. (A) Homozygous CAG42 mice had a reduced body weight by birth up to the age of 21 months, while no change was observed in heterozygous CAG1/CAG42 mice in comparison to wild-type littermates (n≥14 per genotype). (B) Motor coordination was tested on an accelerating rotarod apparatus. An initially increased locomotor performance at 6 weeks of age in the CAG42 mice was replaced by normal activity at 3, 6 and 12 months. At 18 and 21 months age, CAG42 mice showed impaired motor coordination (until 18 months: n≥14 per genotype, 21 months: n≥6 per genotype).

To investigate changes in body weight which frequently accompany neurodegenerative diseases and in particular SCA2, littermate mice with homozygous mutant or wild-type genotypes (Figure S3) were weighed at regular intervals from postnatal day 10 onwards. Already at the age of 10 days, CAG42 mice had a significant body weight reduction of 19.27% compared to wild-type littermates (p = 0.0002); a reduction of 13–22% remained significant throughout their life span (20 days: p = 0.0005; 6 weeks: p = 0.0015; 3 months: p = 0.0025; 6 months: p = 0.0196; 12 months: p = 0.0141; 18 months: p = 0.0265; 21 months: p = 0.0496). Still, the weight gain was similar in CAG42 and WT. The body weight of CAG1/CAG42 was consistently, but non-significantly less than WT (Figure 2A).

The knock-in mice did not show overt ataxic behaviour during cage life. To assess the development of a cerebellar phenotype in more detail, animals were placed on an accelerating rotarod apparatus and their latency to fall was recorded. At 6 weeks, the first time point tested, the latency to fall was 1.2-fold increased (p = 0.0096) in CAG42 mice in comparison to their wild-type littermates. CAG1/CAG42 mice were performing equally well as wild-types. At 3, 6 and 12 months, both CAG42 and CAG1/CAG42 showed no difference to WT in their performance. At the age of 18 months the CAG42 mice showed a significantly shorter latency to fall (0.68-fold, p = 0.0296) from the rotarod, while at 21 months significance was just missed (0.65-fold reduction, p = 0.062), probably due to the low number of animals remaining alive and available for analyses. Thus, CAG42 at early adult age performed better than WT, but at old age considerably worse in this test paradigm with particular sensitivity for cerebellar dysfunction, reflecting possibly an initial overcompensation followed by late-onset pathology. CAG1/CAG42 mice even at old age still performed similarly well as the WT mice (Figure 2B), in keeping with other reports that mice homozygous for polyQ expansion disease show a stronger phenotype than heterozygous animals. Grip strength remained unchanged, indicating that motor neuron pathology and paralysis are absent (data not shown). In addition, footprint analysis did not show any differences between WT and CAG1/CAG42 or CAG42 mice up to 21 months (data not shown). As ATXN2 triplet repeat expansions in human can also lead to Parkinson syndrome with impaired spontaneous movement, the motor activity of mice in an open field was recorded. Neither horizontal/vertical activity, total distance, movement time, number of movements, number of stereotypy counts, margin/centre distance nor margin/centre time were consistently altered (data not shown). Consequently, there was no motor hyperactivity to explain the reduced body weight of knock-in animals. Since the *Atxn2* knock-out mice show excessive weight, the present data might be interpreted as evidence for a gain-of-function effect of the *Atxn2*-CAG42-knock-in in peripheral tissues. In summary, a permanent reduction of body weight and a late-onset impairment of rota rod performance were the only apparent phenotypes in CAG42 mice.

### The *Atxn2* mRNA Is Stable and Its Expression Is Elevated in Cerebellum and Cortex

**Figure 3:**
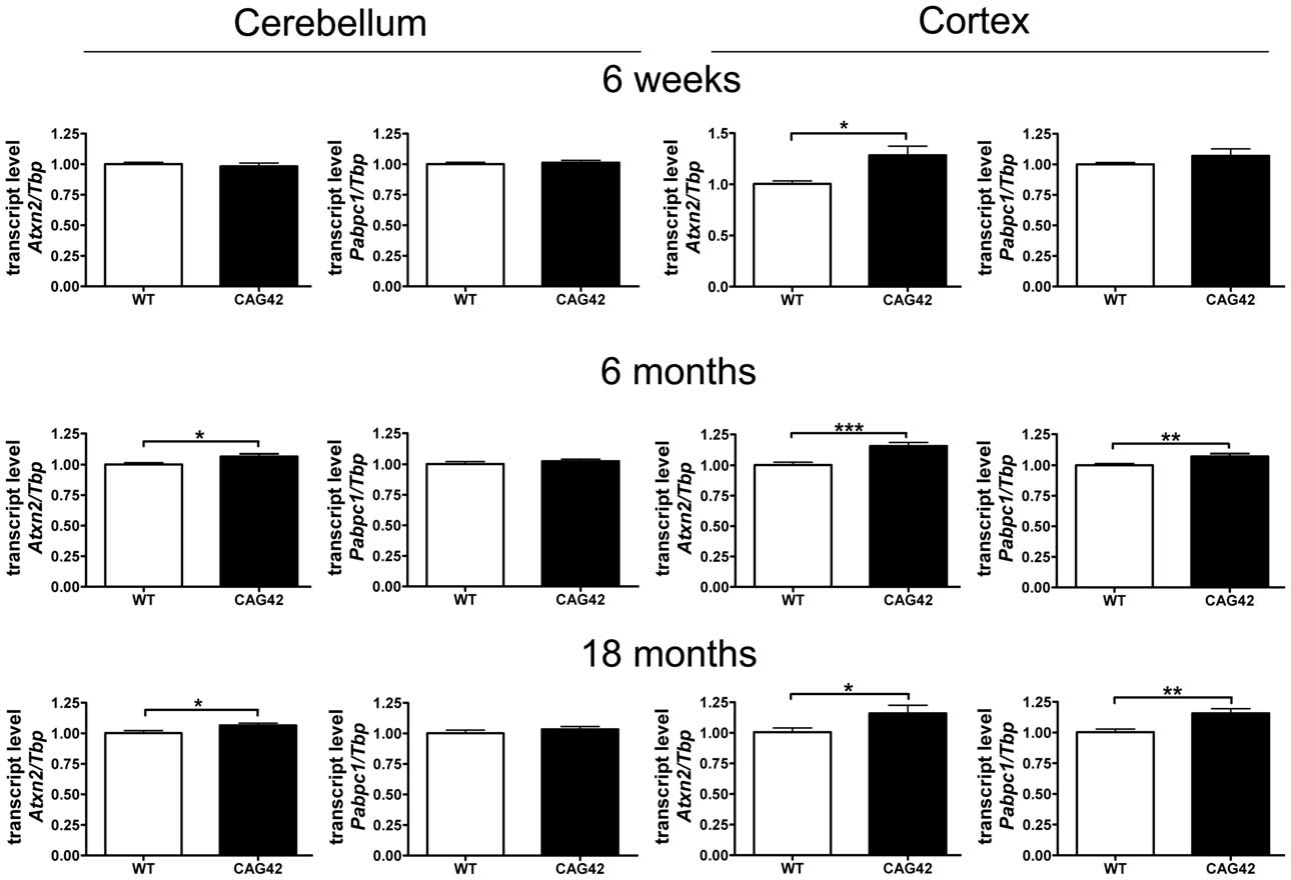
*Atxn2* and *Pabpc1* mRNA levels are elevated in specific ages and areas. In the cerebellum an *Atxn2* mRNA elevation was not detectable until 6 months, whereas the *Pabpc1* transcript levels remained unchanged. In the cortex an *Atxn2* mRNA elevation was already detected by 6 weeks and *Pabpc1* mRNA was upregulated by 6 months (n = 9–16 animals/genotype/tissue).

In order to verify that the homologous recombination event does not interfere with *Atxn2* expression and to assess the stability of the expanded transcript, quantitative real-time RT-PCR (qPCR) was performed in cerebellum and cerebral cortex. While the cerebellar *Atxn2* mRNA levels at 6 weeks of age were unchanged, at 6 months (p = 0.0146) and 18 months (p = 0.0339) they were significantly elevated to 1.07-fold levels. In the cortex, the upregulation of *Atxn2* mRNA expression was already significant at 6 weeks (1.29-fold, p = 0.0101) and stayed significant at 6 months (1.16-fold, p = 0.0005) and 18 months (1.16-fold, p = 0.0474). The transcript levels of the Ataxin-2 interactor *Pabpc1* remained unchanged at all time points in cerebellum. In contrast, in cortex an upregulation of *Pabpc1* mRNA to 1.07-fold (p = 0.0061) and 1.16-fold (p = 0.0028) became apparent at 6 and 18 months, respectively (Figure 3). Thus, a mild but statistically significant elevation of *Atxn2* transcript became apparent during adult life in cerebellum and even earlier in cortex, indicating that the selection marker remaining in the *Atxn2* genomic locus does not impair *Atxn2* transcription and that the presence of a CAG42 repeat in the *Atxn2* transcript does not lead to its instability. Abnormally elevated *Pabpc1* mRNA levels became apparent only in the cortex by 6 months, a noteworthy finding since previous *in vitro* transient transfection studies [41] found the ATXN2 loss-of function to increase PABPC1 levels.

### The Soluble Protein Levels in Old Cerebellum and Cortex Are Reduced for ATXN2 and Altered for PABPC1

**Figure 4:**
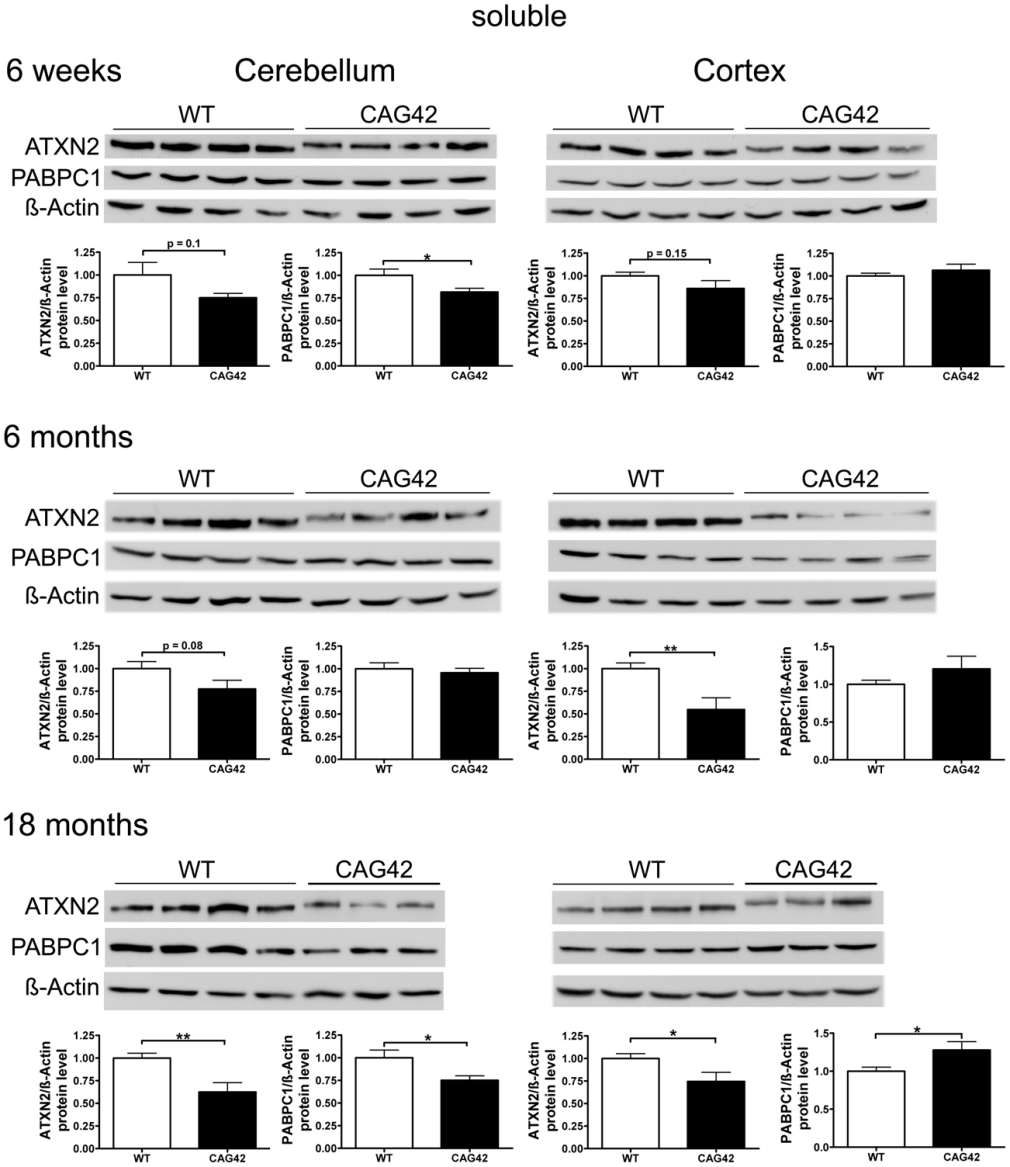
Soluble ATXN2 protein levels are reduced and PABPC1 levels change. In cerebellar tissue, a trend in ATXN2 reduction was observed at 6 weeks and 6 months age, with significance in ATXN2 reduction being reached at 18 months of age. Also, a significant PABPC1 reduction was apparent in 6 weeks and 18 months old CAG42 mice in the cerebellum. In the cortex, a reduction of ATXN2 levels was observed in 6 weeks old animals as a trend and with significance from 6 months onwards. An elevation of PABPC1 levels became significant at 18 months of age (n = 10–12 mice/genotype/tissue).

We investigated the stability and steady-state levels of expanded ATXN2 in extracts of proteins soluble in RIPA extraction buffer. Expanded ATXN2 with 42 glutamines displayed slowed electrophoretic mobility in comparison to wild-type ATXN2 (Figure 4). Both in cerebellum and cortex, a reduction of soluble ATXN2 was detectable in the CAG42 mice. The levels were diminished in the cerebellum consistently at 6 weeks (0.75-fold, p = 0.1008), 6 months (0.77-fold, p = 0.0808) and significantly reduced at 18 months (0.62-fold, p = 0.0037). The reduction of levels in the cortex missed significance at 6 weeks (0.86-fold, p = 0.1553), but became significant both at 6 months (0.55-fold, p = 0.0046) and 18 months (0.75-fold, p = 0.0344). As expected by the elevated *Pabpc1* transcript levels, cortical soluble PABPC1 levels were elevated by 18 months (1.28-fold, p = 0.0293). Curiously, however, cerebellar soluble PABPC1 was significantly reduced already at 6 weeks (0.82-fold, p = 0.034) and again at 18 months (0.75-fold, p = 0.0235) (Figure 4). Thus, a reduction of soluble Q42-ATXN2 protein in spite of elevated transcript levels was a consistent finding in both tissues at all ages, in keeping with a partial loss-of-function and explaining the elevated *PABPC1* transcription in the cortex. However, selectively in the cerebellum PABPC1 soluble protein was also reduced in absence of any compensatory mRNA induction.

### Progressive Insolubility of Q42-ATXN2 and PABPC1

**Figure 5:**
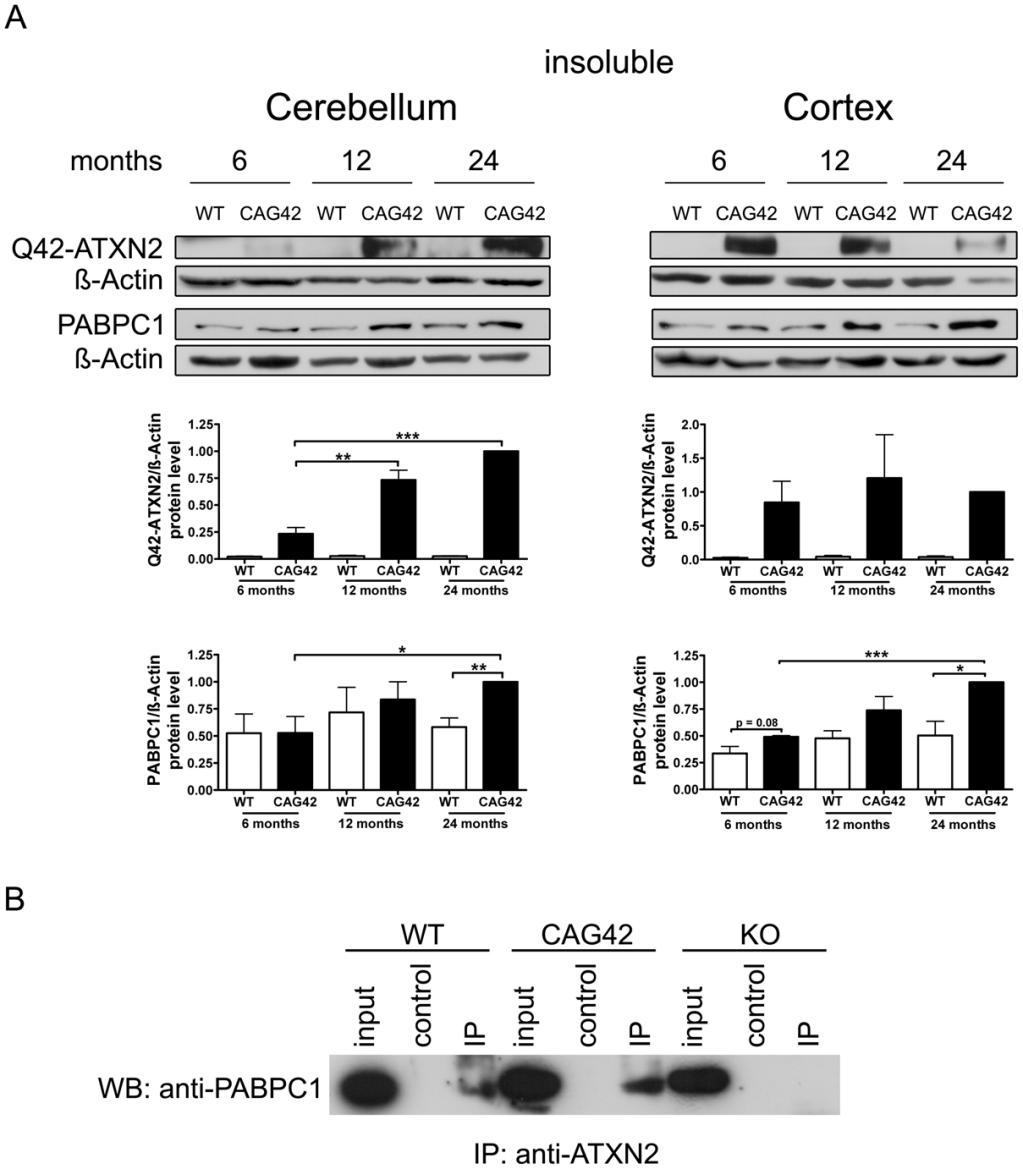
Increased sequestration of PABPC1 by insoluble Q42-ATXN2 with age. (A) In the cerebellum, a progressive insolubility of Q42-ATXN2 from 6 to 12 and 24 months was detectable. The solubility of PABPC1 decreased from 6 to 24 months of age. Insoluble Q42-ATXN2 levels did not change in the cortex, however, the insolubility of PABPC1 increased over time in CAG42 mice. Insoluble PABPC1 levels in WT mice remained stably low in both tissues. (n = 3 mice/genotype/tissue). (B) In the soluble fraction, both wild-type and expanded ATXN2 were able to co-immunoprecipitate PABPC1, with slightly more PABPC1 being pulled down in CAG42 mice.

To test whether increased insolubility of Q42-ATXN2 explains the effects observed, proteins from the pellet after extraction of the soluble proteins were further extracted with a harsher buffer from 6, 12 and 24 months old mice. Detection with the anti-ATXN2 antibody failed, possibly due to epitope masking. Detection with the 1C2 antibody which recognizes polyQ tracts of at least 38 glutamines [45] was successful in revealing the expected band of correct size which was not apparent in wild-type tissue and exclusively appeared in mutant tissue. In the cerebellum a clear increase of insoluble Q42-ATXN2 was detected, which was significant at 12 (3.14-fold, p = 0.0096) and 24 months (4.28-fold, p = 0.0002) in comparison to 6 months Q42-ATXN2. In the cortex, however, an increase was not observed. Also, insoluble PABPC1 protein levels in the cerebellum increased over time and were significantly elevated at 24 months both in comparison to 24 months old WT (1.72-fold, p = 0.0078) and to 6 months old CAG42 tissue (1.9-fold, p = 0.0357). More insoluble PABPC1 was observed in the cortex, a bias which manifested as a trend in 6 months old mice (1.464-fold, p = 0.0812) and became significant by 24 months in comparison to the WT of the same age (1.98-fold, p = 0.02) and also in comparison to 6 months old CAG42 mice (2.036-fold, p<0.0001). In wild-type animals the levels of insoluble PABPC1 levels remained similarly low over time (Figure 5A). Thus, increased insolubility of Q42-ATXN2, particularly in the old cerebellum, appears to sequester PABPC1 into insolubility and may explain the decreased levels of soluble ATXN2 and PABPC1.

In order to test the interaction between ATXN2 and PABPC1 in cerebellum with an independent biochemical method, ATXN2 was immunoprecipitated from wild-type, CAG42 and ATXN2-knock-out tissue and the co-immunoprecipitation of PABPC1 documented in immunoblots. Q42-ATXN2 pulled down slightly more of PABPC1 than wild-type ATXN2 (Figure 5B). Beyond previous reports of association between the two tagged recombinant transfected proteins, our data demonstrate for the first time this interaction between the two endogenous proteins in mammalian tissue. Thus, Q42-ATXN2 is likely to sequester PABPC1 into insolubility through direct interaction.

### ATXN2 and PABPC1 Are Sequestered into Visible Aggregates

**Figure 6:**
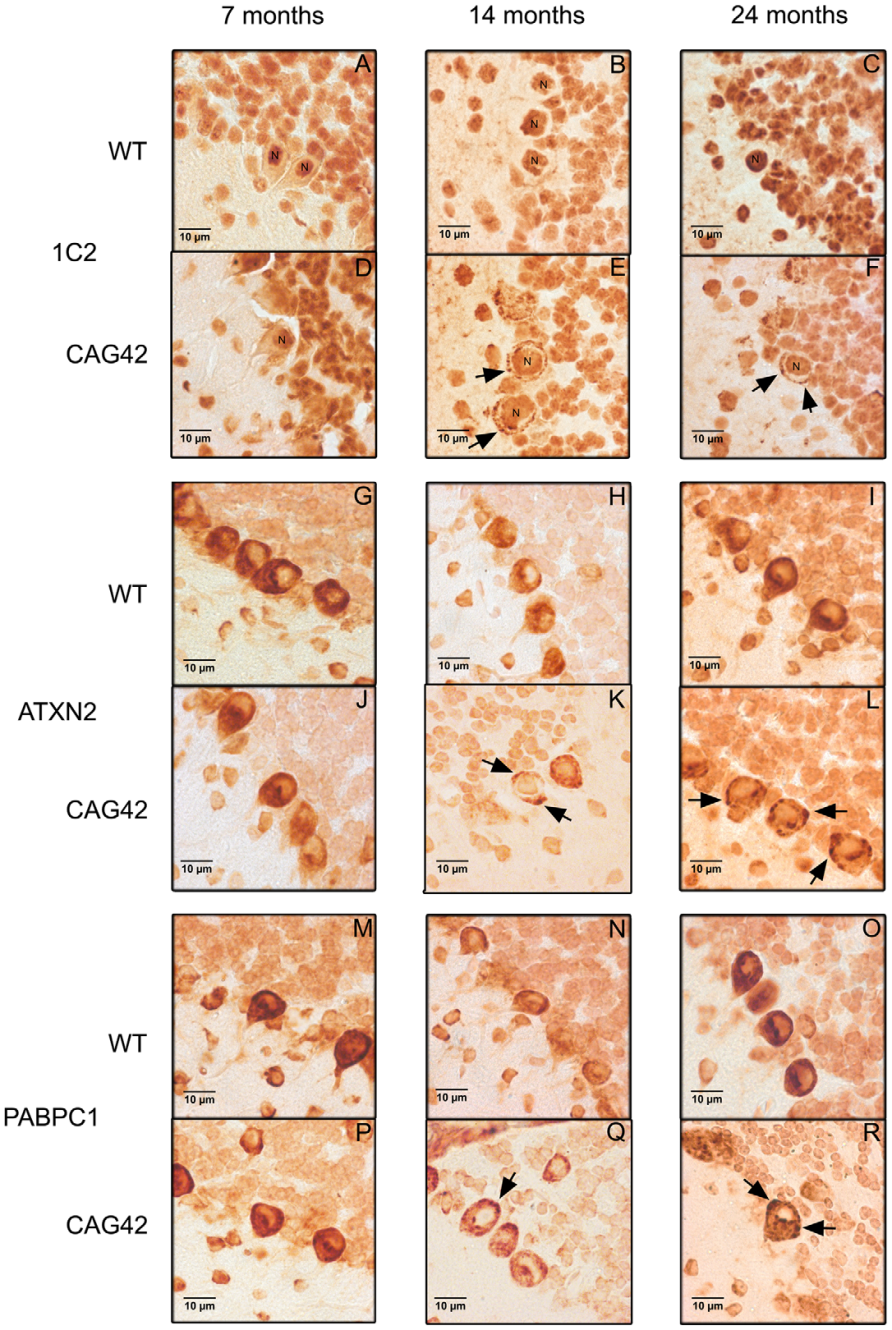
Immunohistochemistry of cerebellar Purkinje cells. At 7, 14 and 24 months age, the Purkinje cells showed a purely nuclear distribution of large polyQ-domains as revealed by 1C2-staining in WT tissue, while an additional granular cytoplasmic staining pattern became apparent at 14 and 24 months in knock-in tissue (A–F). ATXN2 immunoreactivity was diffusely distributed throughout the cytoplasm and concentrated in the perinuclear region in WT tissue, while a more granular appearance was detected discretely at 14 and markedly at 24 months in CAG42 mice (G–L). The expected diffuse cytoplasmic distribution of PABPC1 was documented in WT mice; whereas again a more granular staining pattern in CAG42 mice appeared by 14 months (M–R).

To investigate whether this insolubility process results in the formation of visible cytoplasmic aggregates containing ATXN2, as described in affected neurons during late stages of SCA2 patients, immunohistochemistry on cerebellar brain sections was performed. At all ages tested, 7, 14 and 24 months, 1C2-immunoreactivity was purely nuclear in wild-type mice, while the presence of Q42-ATXN2 resulted in additional cytoplasmic signals of Purkinje neurons, starting discretely by 14 months age and becoming stronger by 24 months (Figure 6A–6F). The specific and selective ATXN2 immunoreactivity of Purkinje neurons could be successfully visualized by an unmasking technique, while remaining undetectable in knock-out tissue (Figure S4). This ATXN2 immunoreactivity of Purkinje cells revealed a diffuse cytoplasmic distribution in WT cerebellum, while it became discretely granular at 14 months in individual Purkinje neurons and markedly granular at 24 months in most Purkinje neurons of CAG42 mice (Figure 6G–6L). In addition, the PABPC1-immunoreactivity was diffusely cytoplasmic in WT mice, but became discretely granular in some Purkinje neurons by 14 months and markedly granular in most Purkinje neurons by 24 months of age (Figure 6M–6R). Nuclear inclusion bodies were never detected. A decrease of the molecular layer thickness or of the Purkinje cell number (data not shown), or a reduction in the intensity of Calbindin immunoreactivity (Figure S5) was not observed even at the age of 24 months, suggesting that a neurodegenerative process is not yet detectable at this age. Thus, the histological analysis supports the concept of progressive insolubility and aggregation of Q42-ATXN2 as well as PABPC1 in the cerebellar Purkinje neurons which are the prominent site of pathology in human SCA2.

### Insoluble Normal and Expanded ATXN2 *In Vitro* Drives Endogenous PABPC1 into Insolubility

**Figure 7:**
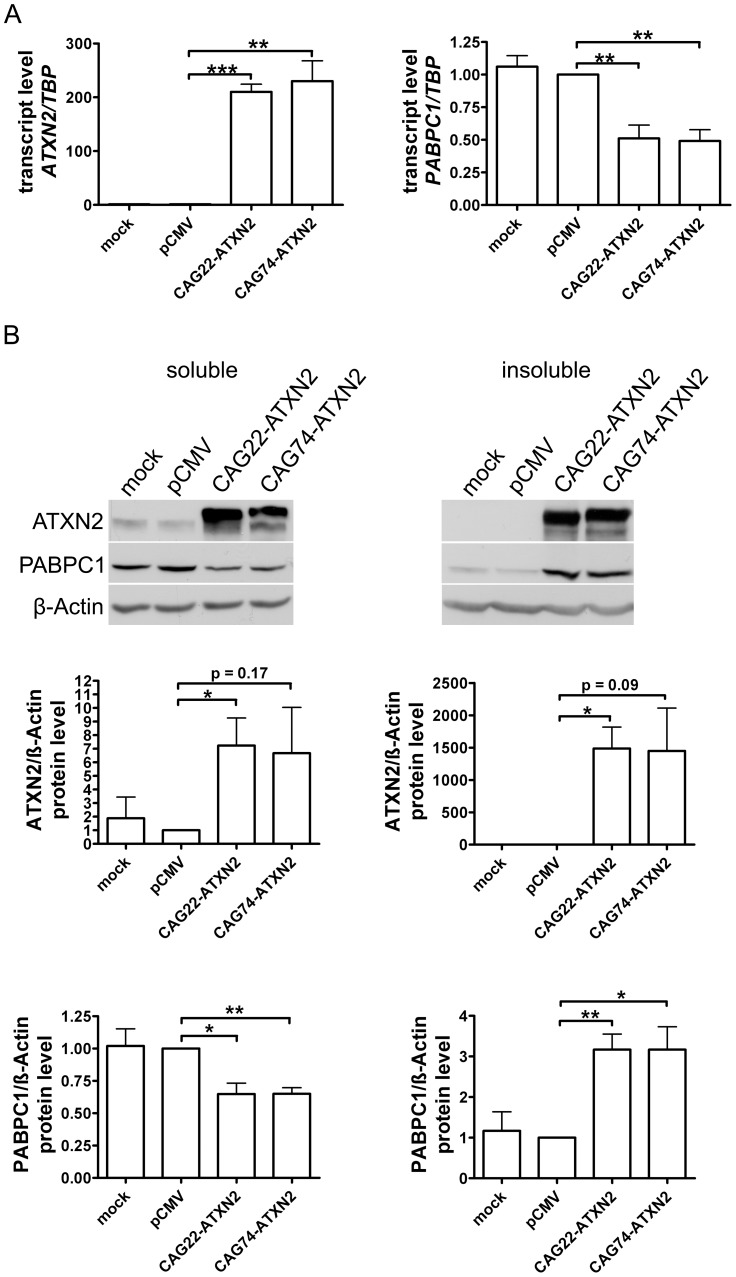
Normal and expanded insoluble ATXN2 drives PABPC1 into insolubility. (A) Overexpression of CAG22-ATXN2 and CAG74-ATXN2 led to significantly decreased *PABPC1* transcript levels. (B) Overexpressed Q22-ATXN2 or Q74-ATXN2 reduced endogenous soluble PABPC1 levels, whereas endogenous insoluble PABPC1 levels were significantly increased in the presence of insoluble ATXN2 (n = 3).

To confirm *in vitro* that insoluble expanded ATXN2 influences the PABPC1 solubility, HeLa cells were transfected transiently with CAG22-ATXN2 and CAG74-ATXN2 constructs. Successful overexpression was verified by qPCR and Western blot. Over 200-fold elevated *CAG22-ATXN2* and *CAG74-ATXN2* mRNA levels were accompanied by reduced *PABPC1* transcript levels (approximately 0.5-fold, p = 0.0087 and p = 0.0041) (Figure 7A), while at the protein level (Figure 7B) a 7.2-fold (p = 0.0374) and 6.7-fold (p = 0.1667) excess of soluble Q22-ATXN2 or Q74-ATXN2 was found, accompanied by a reduction of endogenous soluble PABPC1 levels to 0.65-fold (p = 0.0138 and p = 0.002). To assess the insoluble fractions of both proteins, the pellet after the extraction of soluble proteins was dissolved and re-extracted with more detergents. Overexpressed Q22-ATXN2 and Q74-ATXN2 were strongly detectable, while endogenous ATXN2 in the empty vector control transfection was practically undetectable. Endogenous PABPC1 levels in the insoluble protein fraction were increased to 3.2-fold after both Q22-ATXN2 (p = 0.0048) and Q74-ATXN2 (p = 0.0184) overexpression. These data corroborate our previous tissue data that insoluble ATXN2 of either normal or expanded size sequesters its interactor protein PABPC1 into insolubility and may thus contribute to the reduction of soluble PABPC1 levels. They are also in keeping with the previous *in vitro* reports [41] that the increase in soluble ATXN2 levels leads to reduced PABPC1 expression levels.

### Microarray Transcriptome Profiling at Medium and Advanced Age Documents Selective Induction of FBXW8 in Old Cerebellum

Attempting a survey of the alterations in mRNA processing underlying this progressive pathology, we used cerebellum, brainstem and liver tissue from presymptomatic 6 months and symptomatic 18 months old WT and CAG42 mice (3 versus 3 animals per young group, 4 versus 4 per old group) to perform Affymetrix microarray transcriptome studies at a genome-wide level. At the age of 6 months, statistical analysis with correction for multiple testing after Benjamini-Hochberg demonstrated that no mRNAs showed dysregulated levels with significance in cerebellum and brainstem, while only one gene was differentially regulated in liver. This gene *Cyp4a14*, is represented only by one oligonucleotide spot on the microarray. The Cytochrome P-450 enzyme family has a known role in cholesterol biosynthesis and the Cyp4a14 induction is a known response of liver tissue to altered fat content [46]. Since fatty liver is a feature already documented and molecularly investigated in ATXN2-KO mice [47], we did not investigate this finding further. At the age of 18 months, altered regulation was significant after Benjamini-Hochberg correction for 20 genes in cerebellum, 14 genes in brainstem and 30 genes in liver. Among those, the dysregulation of 5 genes was recognized consistently by more than one oligonucleotide microarray spot (Table 1). Upon independent validation of the findings by qPCR with commercial Taqman assays, the dysregulations of *Acat1*, *Ifi27l1* and *Lgals1* could not be reproduced in tissue from different animals; in contrast, a significant downregulation of *Adam1a* in cerebellum to 0.71-fold (p = 0.0007) and brainstem to 0.73-fold (p = 0.001) as well as a significant upregulation of *Fbxw8* to 2.38-fold (p<0.0001) in cerebellum were confirmed in this approach. *Adam1a* encodes a disintegrin/metalloprotease and has an established role for spermatogenesis and fertilization [48]. Thus, its dysregulation might relate to the reduced fertility observed in ATXN2-KO mice [47]. *Fbxw8* encodes a WD-40 domain containing member of the F-box protein family, which are substrate-recognition mediators within a SCF (SKP1-CUL1-F-box)-type ubiquitin-E3-ligase complex involved in the tagging of phosphorylated target proteins to destine them for degradation [49].

**Table 1:** Differentially regulated mRNAs in CAG42 mice at the age of 18 months.

Symbol	Genomic locus	Spot ID	Cerebellum	Brainstem	Liver
			Fold-change (p-value)	Fold-change (p-value)	Fold-change (p-value)
***Acat1***	9 C–D	1451271_PM_a_at	—	—	**−1.42** (0.0101)
		1424183_PM_at	—	—	**−1.50** (0.0071)
		1424182_PM_at	—	—	**−1.58** (0.0052)
***Adam1a***	5 F	1443378_PM_s_at	**−1.77** (0.0009)	**−1.47** (0.0009)	—
		1427790_PM_at	**−1.89** (0.0071)	—	—
***Fbxw8***	5 F	1426944_PM_at	**2.55** (0.0077)	—	—
		1436732_PM_s_at	**2.51** (0.0095)	—	—
***Ifi27l1***	12 E	1452956_PM_a_at	—	—	**3.27** (0.0273)
		1454757_PM_s_at	—	—	**3.20** (0.0213)
***Lgals1***	15 E	1419573_PM_a_at	—	—	**2.27** (0.0165)
		1455439_PM_a_at	—	—	**2.14** (0.0459)

Genes with significant dysregulation and consistency between more than one oligonucleotide spot are shown (n = 4 mice/genotype).

### FBXW8 Protein Upregulation Found in Old Cerebellum Reduces the Insoluble Levels of Expanded ATXN2 in a Human Cell Line

**Figure 8:**
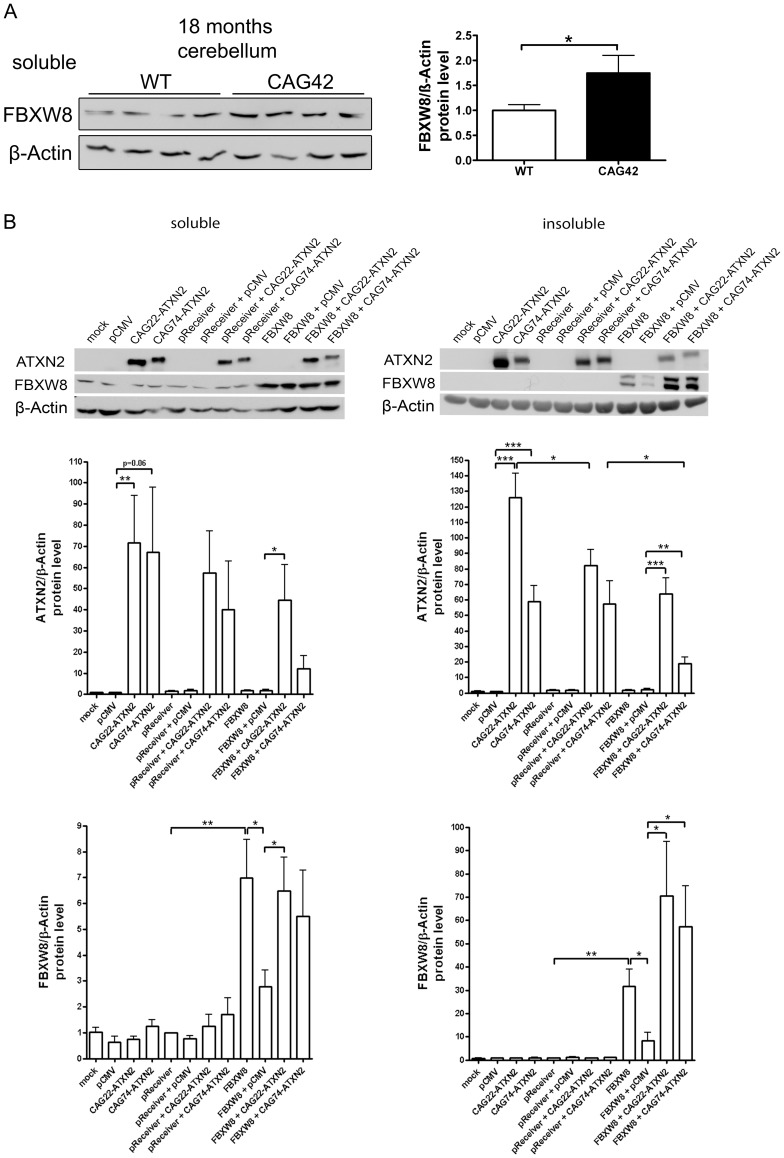
Elevated FBXW8 diminishes specifically insoluble expanded ATXN2. (A) The protein levels of FBXW8 were significantly upregulated in the cerebellum of 18 months old CAG42 mice compared to wild-type (n = 9–11 mice/genotype). (B) Soluble levels of Q22-ATXN2 were unchanged after FBXW8 overexpression, while for Q74-ATXN2 a slight reduction was apparent. With respect to insoluble levels, Q22-ATXN2 was again not influenced by FBXW8 overexpression, while the Q74-ATXN2 reduction became significant. FBXW8 levels were significantly increased by its overexpression both in the soluble and insoluble fraction (n = 7).

To assess whether *Fbxw8* induction on transcript level results in elevated FBXW8 protein levels, Western blots in 18 months old cerebellum of WT and CAG42 mice were performed (Figure 8A). A significant upregulation of FBXW8 to 1.75-fold (p = 0.043) in CAG42 mice was documented. Thus, elevated Fbxw8 mRNA and protein levels characterize the cerebellar tissue of old CAG42 mice.

To further examine the questions whether the selective upregulation of *Fbxw8* in old CAG42 cerebellum reflects a compensatory cellular response to pathology and whether this is an efficient regulation to degrade expanded ATXN2 also in humans, we assessed the effects of elevated FBXW8 protein on the soluble and insoluble levels of human ATXN2 *in vitro*. HeLa cells were transiently single and double transfected with CAG22-ATXN2, CAG74-ATXN2 and FBXW8 (Figure 8B). Transfection only with CAG22-ATXN2 or CAG74-ATXN2 in the soluble phase led to a 72-fold increase of Q22-ATXN2 (p = 0.0098) or a 67-fold increase of Q74-ATXN2 (p = 0.056) protein compared to the control vector pCMV. Additional expression of the control vector pReceiver did not reduce Q22-ATXN2 or Q74-levels significantly (57-fold, p = 0.64, and 40-fold p = 0.51). A further overexpression of FBXW8 reduced Q74-ATXN2-levels by a factor 3.3 (p = 0.23), while it did not markedly change Q22-ATXN2 levels. In the insoluble fraction, transfection led to elevated Q22-ATXN2 (126-fold, p<0.0001) and Q74-ATXN2 (59-fold, p<0.0001) levels. Additional expression of the empty pReceiver vector reduced Q22-ATXN2 levels (82-fold, p = 0.036) and did not alter Q74-ATXN2 levels (57-fold, p = 0.93). After double transfection with FBXW8 and Q22-ATXN2 or Q74-ATXN2, the levels of ATXN2 were again significantly elevated (64-fold, p = 0.0002 and 18-fold, p = 0.005, respectively) compared to the FBXW8+pCMV control. This elevated level was significantly less high for expanded ATXN2 in comparison to the control double transfection with pReceiver and CAG74-ATXN2 (p = 0.028). Control immunodetection of the FBXW8 levels demonstrated the successful overexpression to result in detectable upregulation in soluble and insoluble fractions, and a curious increase of FBXW8 insoluble protein levels in the presence of ATXN2. These *in vitro* human data indicate that the upregulation of FBXW8 reduces the insoluble levels selectively of expanded ATXN2. Analogous double transfection experiments showed that also the insoluble fraction of Q41-ATXN2 becomes slightly reduced by FBXW8, but this effect did not become significant in three independent experiments (Figure S6).

## Discussion

We have generated and characterized the first knock-in mouse modelling Spinocerebellar Ataxia Type 2. Since SCA2 is a late-onset progressive disease with selective vulnerability of the cerebellar Purkinje neurons, it is interesting that similar temporal dynamics were observed for the insolubility of ATXN2 and for the accompanying sequestration of PABPC1 in the knock-in cerebellum. The progressively granular immunoreactivity of ATXN2 and PABPC1 in Purkinje neuron immunohistochemistry supports the concept of aggregate accumulation, and the *in vitro* and Co-IP data confirm that excess ATXN2 becomes insoluble and recruits its interactor PABPC1 into insolubility. Progressively granular immunoreactivity of both proteins becomes apparent in increasing numbers of cerebellar Purkinje neurons even before the onset of the selectively cerebellar motor deficits. Probably this pathological process of aggregation corresponds to the toxic gain-of-function which was shown to drive disease progression in other polyQ disorders. A gain-of-function of ATXN2 might also underlie the reduced weight of the knock-in animals, since ATXN2 knock-out mice are known to have excessive weight [47]. Specifically in cerebellar tissue, the insolubility and sequestration led to a deficiency in soluble PABPC1 levels, an effect that might result in impaired RNA processing as well as protein synthesis [50] and therefore possibly critical for the Purkinje neurons with their large ribosomal machinery. Indeed, these molecular abnormalities correlate temporally with the late-onset appearance of motor deficit.

The depletion of PABPC1 was shown to prevent global protein synthesis and to promote cell death in mammalian cells [50]. PABPC1 binds to the 3′ poly(A) tail of mRNAs to mediate their circularization which precedes ribosome recruitment and translation initiation [51]. Furthermore, PABPC1 is important for the poly(A) shortening and nonsense-mediated decay of mRNAs in the cytoplasm [52], [53]. During the formation of stress granules, PABPC1 and ATXN2 relocalize there together with translationally repressed mRNA complexes [41]. Furthermore, ATXN2 could play a role in mRNA splicing, since it contains Lsm domains and since it interacts with A2BP1/Fox1 which is a well established splicing modulator [54], [55]. Finally, ATXN2 might modulate transcription itself, since it was shown to relocalize to the nucleus and control its own expression [56]. Thus, there are multiple ways how ATXN2 could modulate RNA processing, and indeed the confirmed role of ATXN2 expansions in interaction with other RNA binding proteins for motoneuron degeneration diseases [5], [6], [57] indicates this to be a crucial aspect of ATXN2 effects. Our present data support the concept that reduced levels of PABPC1 are associated with the cerebellar vulnerability in SCA2, and that the pathogenesis might have similarities to the altered mRNA processing in ALS, FXTAS and SMA [58]. Unfortunately, the rates of mRNA translation and deadenylation cannot be directly quantified in tissue, so further mechanistic analyses will have to rely on *in vitro* studies.

It is interesting to note that in the cerebellum the progressive PABPC1 insolubility and the ATXN2 gain-of-function led to decreased soluble PABPC1 levels that did not elicit a compensatory upregulation of PABPC1 transcript levels. This may simply be due to a maximal PABPC1 expression level in cerebellar cells that cannot be enhanced further. In contrast, in cortex tissue a similarly progressive PABPC1 sequestration appeared overcompensated in the face of elevated levels of PABPC1 transcript and soluble protein. We speculate that the cause of this upregulated cortical PABPC1 expression lies in a response to the deficiency in soluble ATXN2, since PABPC1 levels were previously found *in vitro* to correlate indirectly with ATXN2 levels [41]. This partial loss of soluble ATXN2 was observed in spite of elevated mRNA levels in a quite constant manner in all ages and brain regions. This is particularly interesting, since in other polyQ neurodegenerative diseases some minor symptoms have clearly been attributed to a partial loss-of-function mechanism. For example, polyQ expansions in the androgen receptor lead to a partial loss-of-function of this protein with subsequent testicular feminisation of male patients, apart from the atrophy of spinobulbar motoneurons which contain polyQ aggregates [59].

Our investigation into consequences of ATXN2 expansions for mRNA levels in a genome-wide transcriptome survey demonstrated a surprising scarcity of effects, with the notable exception of the highly significant, reproducible and specific dysregulation in the expression of two ATXN2 neighbour genes, *Adam1a* and *Fbxw8*. The murine *Atxn2* gene lies on chromosome 5 F (position 122,162–122,265 kbp), with *Adam1a* (position 121,969–121,972 kbp) being positioned at a distance of ∼190 kbp from *Atxn2*, and *Fbxw8* (position 118,515–118,606 kbp) being located at a distance of ∼3556 kbp from *Atxn2*. Three different hypotheses may explain these transcription effects with specificity to the *Atxn2* locus. Firstly, it is conceivable that the knock-in targeting approach has modified the *Atxn2* locus, but the facts that the selection markers were almost completely removed and that this locus effect was not detectable in the transcriptome data at 6 months age argue against this notion. Secondly, notions have been discussed that the abnormal DNA structure of a (CAG)-expansion could exert a progressive influence on chromatin structure [60]; indeed, polyglutamine expansions of the SCA7 disease protein, which is a component of a histone acetyltransferase complex, was shown to result in aberrant chromatin [61]. However, such effects were not confined to the surrounding locus and represented random toxic side-effects on unrelated proteins rather than an influence on the disease protein and its function. Thirdly, it is possible that the genes that surround the *Atxn2* locus are interacting in a common pathway and that feedback-mechanisms regulate their expression in dependence on ATXN2 function. Our *in vitro* data that *Fbxw8* upregulation may selectively reduce the levels of insoluble, expanded ATXN2 argues in favour of the latter hypothesis. A compensatory cellular effort that degrades the expanded and toxic ATXN2 and counteracts its gain-of-function would help to minimize the alterations of interactors with downstream consequences, thus postponing the onset of pathology; therefore FBXW8 represents a promising molecular target for neuroprotective approaches. FBXW8 was reported to control growth in parallel to modulating insulin-like growth factor binding protein 1 levels [62]. As FBXW8 null mice are growth retarded [62], the induction of FBXW8 at old age in the knock-in tissue could again be interpreted as an effort to compensate the weight reduction caused by CAG42-ATXN2. Furthermore, FBXW8 modifies neuronal dendrite formation [63]. It is also noteworthy that ATXN2 was observed to interact with the neuroprotective ubiquitin E3 ligase PARKIN [64], and that FBXW7 (SEL-10) is also known to interact with PARKIN [49]. It goes beyond the scope of this manuscript to test whether FBXW8 or other members of this protein family may also contribute to the degradation of other polyglutamine-expansion disease proteins like huntingtin and ataxin-1. Overall, the early induction of *Cyp14a4* and the later induction of *Fbxw8* are probably part of the cellular stress response. In order to study further progression of the neurodegenerative process beyond such early compensatory efforts, and to understand later SCA2 and ALS stages, ATXN2 knock-in mice with larger CAG expansions or with CAA interruptions may have to be studied.

In conclusion, our first report of a knock-in mouse with CAG42 to model SCA2 confirms that progressive insolubility of ATXN2 particularly in the cerebellum is the molecular feature which correlates best with the late-onset motor incoordination. The subsequent sequestration of the interactor protein PABPC1 into insolubility provides a first insight from the analysis of brain tissue for the recent focus how SCA2 and ALS pathogenesis might affect RNA processing [65], [66]. The cerebellar tissue differed from cortical tissue by the lack of a compensatory upregulation of PABPC1 expression. Specifically in cerebellar tissue at old age, an upregulation of *Fbxw8* levels was observed, which may contribute to counteract the ATXN2 gain-of-function according to our human *in vitro* studies. These findings may lead us to understand the tissue specificity in this disease process. We believe that this knock-in mouse will constitute a valuable tool for pathogenesis research in early stages of SCA2 and possibly also ALS, given the scarce availability of human autopsy brain tissues. Although the ATXN2 triplet repeat is transmitted stably across generations in mouse in contrast to humans, this may be an advantage for the long term comparability of data from this knock-in model.

## Materials and Methods

### Generation of *Atxn2*-CAG42-Knock-In Mice

*Atxn2*-CAG42-knock-in mice were generated by modifying the previously described pKO-*Sca2*-vector [47]. Exon 1 was deleted via the unique restriction sites SgrAI and Eco47III and replaced by a synthesized exon 1 fragment containing the CAG42-repeat (custom-made by Geneart, Regensburg) at the amino acid position glutamine 156, resulting in the targeting vector NOW1-HR. The sequence verified vector was electroporated into 129Sv/Pas ES cells to allow for homologous recombination at the endogenous *Ataxin-2* locus. Its integration was verified using PCR. The correct 3′ integration was verified using a forward primer hybridizing in the neomycin selection cassette and a reverse primer downstream of the targeting vector homology sequence. Verification of the correct 5′ integration was achieved with a primer pair flanking the loxP site. The forward primer was located upstream of the long homology arm of the targeting vector and the reverse primer hybridized upstream of the distal loxP site within the 5′ homology arm of the targeting vector. Additionally, the integration of the CAG repeat insertion was verified using primers flanking the CAG repeat. Further validation was performed by Southern blot analysis (Figure S1). Removal of the FRT flanked neomycin resistance cassette was performed via Flp-mediated excision and its success again verified using PCR and Southern blot analysis (data not shown). Correctly targeted ES cell clones were injected into C57BL/6 blastocysts. This work was outsourced to Genoway (Lyon, France). For primer sequences see Tables S2, S3, S4, S5.

### Genotyping of the *Atxn2*-CAG42-Knock-In Mouse

DNA from tail biopsies was isolated and the genotyping PCR was performed. TaKaRa LA Taq-Polymerase (Takara Bio Inc., Japan) was used to amplify the neomycin cassette excised locus with the primer pair NOW1-K2 5′-TGAGTTGACTCCACAGGGAGGTGAGC-3′ and NOW1-H2 5′-CCATCTCGCCAGCCCGTAAGATTC-3′ flanking this site. The conditions were: initial denaturation at 94°C-3′, followed by 30 cycles of 94°C-15″ denaturation, 68°C-4′ annealing and elongation and a final elongation step at 68°C-9′. The wild-type (WT) allele is predicted to yield an amplification product of 793 bp and the knock-in (CAG42) allele one of 984 bp, while heterozygous (CAG1/CAG42) mice show products of both sizes (Figure S3).

### Animals

Mice were housed in accordance with the German Animal Welfare Act, the Council Directive of 24 November 1986 (86/609/EWG) with Annex II and the ETS123 (European Convention for the Protection of Vertebrate Animals) at the FELASA-certified Central Animal Facility (ZFE) of the Frankfurt University Medical School. Mice were backcrossed from a mixed 129Sv/Pas×C57BL/6 for at least 8 generations into the C57BL/6 strain. Littermates derived from heterozygous matings were used for all experiments.

### Body Weight and Behavioural Observations

For behaviour observations, male and female mice were used in similar proportions. Mice were weighed before behavioural testing. The motor performance of mice was assessed using an accelerating rotarod apparatus (Ugo Basile, Comerio, Robert & Jones, model 7650). Mice from three founder lines were placed at different ages on the accelerating rod (from 4–40 rpm) and latency to fall was recorded during the 5 minute trial. Grip strength was assessed by measuring the peak force of the fore limbs in 10 trials per mouse on an electronic grip strength meter (TSE, Bad Homburg). Footprints were evaluated by painting the hind limbs of mice with a non-toxic ink. The mice were allowed to walk through a paper lined tunnel (height: 6 cm×width: 9 cm×length: 40 cm) and step length, gait width, alternation coefficient and linear movement were analyzed on the basis of the footprints as described previously [67]. The spontaneous motor activity of the mice was recorded by a Versamax animal activity monitor (Accuscan, Columbus). The mouse was placed into the 20×20 cm arena and its activity recorded during the 5 minute trial.

### Verification of the CAG Repeat Length

The determination of the CAG repeat length was done using fragment analysis. The CAG repeat was amplified with TaKaRa LA Taq-Polymerase (Takara Bio Inc., Japan) from DNA from tail biopsies using a 5′-FAM-labeled forward primer 5′-CCCCGCCCGGCGTGCGAGCCGGTGTAT-3′ and the reverse primer 5′-CGGGCTTGCGGCCAGTGG-3′ under the following conditions: 96°C-4′, followed by 38 cycles of 94°C-1′, 60°C-1′ and 72°C-1′ with a final elongation step at 72°C-7′. The determination of the fragment length was outsourced to GENterprise GENOMICS (Mainz, Germany). Peak Scanner Software 1.0 (Applied Biosystems) was used to determine the exact PCR product size using the GS500 size standard.

### RNA Isolation and Expression Analysis

After cervical dislocation the brain was removed, cerebellum and cortex were dissected and quickly frozen in liquid nitrogen. RNA extraction from tissue was performed with Trizol reagent (Invitrogen) and from cells using the RNeasy Mini Kit (Qiagen) according to manufacturers' instructions. For expression studies, 1 µg of total RNA was digested with DNaseI Amplification Grade (Invitrogen) and reversely transcribed using SuperScript III Reverse Transcriptase (Invitrogen). Expression levels were investigated with a StepOnePlus Real-Time PCR System (Applied Biosystems). cDNA from 25 ng RNA were used in each PCR reaction with the following TaqMan Assays (Applied Biosystems): *Atxn2* (Mm01199894_m1, Hs00268077_m1), *Pabpc1* (Mm00849569_s1, Hs00743792_s1), *Acat1* (Mm00507463_m1), *Adam1a* (Mm02581738_s1), *Fbxw8* (Mm00554876_m1), *Ifi27l1* (Mm00835449_g1), *Lgals1* (Mm00839408_g1) and *Tbp* (Mm00446973_m1, Hs99999910_m1) as endogenous control. The PCR conditions were 50°C-2′, 95°C-10′ followed by 40 cycles of 95°C-15″ and 60°C-60″. The gene expression data were analyzed according to the 2^−ΔΔCt^ method [68].

### Western Blot

50 mg of tissue were homogenized with a motor pestle in 10 vol. RIPA buffer [50 mM Tris-HCl (pH 8.0), 150 mM NaCl, 1 mM EDTA, 1 mM EGTA, 1% Igepal CA-630 (Sigma), 0.5% sodium deoxycholate, 0.1% SDS, 1 mM PMSF and one tablet Complete Protease Inhibitor Cocktail (Roche)] followed by 15 minutes incubation on ice. After centrifugation at 16,000×g at 4°C for 20 minutes, the supernatant (soluble fraction) was preserved and either 1/2 vol. Urea/SDS-buffer [8 M Urea, 5% SDS, 1 mM PMSF, Complete protease inhibitor cocktail (Roche)] for tissue extraction or 1/2 vol. 2× SDS-lysis buffer [137 mM Tris-HCl, pH 6.8; 4% SDS; 20% glycerol] for cell extraction was added to the pellet, sonicated and centrifuged at full-speed for 10 minutes. The supernatant represented the insoluble fraction. Extracted proteins were processed directly without freezing them. Protein concentration was determined using the BCA protein assay kit (Thermo Scientific). 10 or 20 µg of proteins were loaded and separated on 8% polyacrylamide gels and transferred to PVDF membranes. The membrane was blocked in 5% milk powder, incubated with primary antibodies against Ataxin-2 (1∶500, BD Transduction Laboratories), PABPC1 (1∶1000, Abcam), 1C2 (1∶1000, Millipore), FBXW8 (1∶750, Sigma) and β-Actin (1∶10,000, Sigma) and visualized using ECL method (Pierce). Densitometric analysis was carried out using the ImageJ software.

### Immunohistochemistry

The cerebellum of perfused (4% PFA) mouse brains was cut on a microtome into 5 µm thick sagittal sections. For antibody staining, sections were immersed for 30 minutes in 10% methanol, 3% H_2_O_2_ and 50 mM Tris (pH 7.6) and subsequently washed for three times in Tris. Sections were blocked for 90 minutes with 0.25% Triton X, 0.1 M D-Lysine and 10% Tris-BSA. Blocking before Ataxin-2 staining was modified using goat-serum in 0.1 M PBS. Incubation with the primary antibodies against Ataxin-2 (BD Transduction laboratories, 1∶50), PABPC1 (Cell Signaling, 1∶50), 1C2 (1∶1000, Millipore) or Calbindin (1∶3000) lasted 18 h at room temperature. The biotinylated secondary antibody (1∶200) was applied for 90 minutes followed by incubation for 2 h in the avidin-biotin-peroxidase complex (1∶100 in Tris, ABC-Elite, Vector Laboratories). Finally, sections were incubated in Tris with 0.07% DAB and 0.001% H_2_O_2_. For Ataxin-2 staining sections were pretreated for 30 sec at 125°C in bull's eye decloaker solution in a decloaker chamber (Decloaker, Biocare Medical); for PABPC1 staining, sections were pretreated with citrate buffer; for 1C2 staining the sections were incubated in the microwave 3 times for 10 minutes in Tris (pH 9.0), followed by a 3 minute incubation step in 98% formic acid; for Calbindin staining the sections were pretreated with trypsin. Determination of the molecular layer thickness, Purkinje cell number and intensity of Calbindin immunoreactivity were done by ImageJ in 2 mice/genotype with at least 3 sections/animal and at least 10 microscopic fields/section.

### Co-Immunoprecipitation

Approximately 50 mg of cerebellar tissue from 6 months old animals were lysed in 10× vol. lysis buffer (10 mM HEPES, 10 mM KCl, 5 mM MgCl_2_, 0.1% Igepal CA-630 (Sigma), protease inhibitors), incubated for 10 minutes at 4°C and subsequently centrifuged for 20 minutes at 16,000 g. 200 µg of soluble proteins derived from the supernatant were incubated overnight under rotation with anti-ATXN2 antibody. Beads (Protein A/G-agarose, Santa Cruz) were pre-treated overnight with washing buffer (0.2 NaCl, 1% gelatine, 0.05% NaN_3_, 50 mM Tris, 0.1% Triton) to avoid unspecific protein binding. Antibody-protein complexes were precipitated with beads under rotation for one hour at 4°C. Beads were sedimented by centrifugation and washed for 4 times in PBS including protease inhibitors and then subjected to Western blotting.

### Cell Culture

HeLa cells were cultured in MEM medium (Invitrogen) with 10% FCS, 1% NEAA and 1% HEPES and seeded the day before the experiment. 1 µg of the plasmids pCMV-Myc, ATXN2(CAG22)-Myc, ATXN2(CAG74)-Myc [38], FBXW8-pReceiver-M55 (with Cherry-tag) and pReceiver-M56 (with Cherry-tag) (Imagenes) were used for the transfection with Effectene Transfection Reagent (Qiagen) according to manufacturer's protocol.

### Microarray Transcriptome Analysis

Cerebellum, brainstem and liver tissue from 6 and 18 months old WT and CAG42 mice were sent to the MFT Services (Tübingen, Germany) for analysis. The RNA was isolated, its quality documented (one young CAG42 liver sample had to be eliminated from analysis) and 100 ng of total RNA was amplified with the Affymetrix 3′ IVT Express Kit, labeled and hybridized onto the mouse specific MOE430 2.0 Gene Chip (Affymetrix, Santa Clara, CA, USA) that detects 39,000 transcripts and variants corresponding to 34,000 mouse genes. Scanning of the arrays was performed in the Gene Chip Scanner 3000 and the raw data obtained with the AGCC 3.0 software. Further analysis was done using the Bioconductor package (www.bioconductor.org). RMA normalization was applied and calculation of differentially expressed transcripts was done by compiling a linear model. F-statistics was applied (empirical Bayes model) and the p-values obtained were further corrected for multiple testing with the “Benjamini-Hochberg” test. P-values<0.05 were considered significant. Genes that were detected within their exons and that were dysregulated consistently according to more than one spot were prioritized for further analysis. The original data from 18 months old animals have been deposited in NCBI's Gene Expression Omnibus [69] and are accessible through GEO Series accession number GSE39640 (http://www.ncbi.nlm.nih.gov/geo/query/acc.cgi?acc=GSE39640) (NCBI tracking system #16608301).

### Statistical Analysis

The GraphPad Prism software version 4.03 (2005) and MS Excel 2007 (Microsoft) were used to perform unpaired Student's t-test. Error bars indicate SEM. Values p<0.05 were considered significant and marked with asterisks p<0.05 *, p<0.01 **, p<0.001 ***.

## Supporting Information

Figure S1 Southern blot strategy for the detection of homologous recombination at the *Atxn2* locus. Exon 1 is symbolized by a hatched rectangle and the CAG repeat by a light grey rectangle. FRT sites are represented by grey double triangles and loxP sites by a single triangle. The expected DNA fragment sizes for the 5′ Southern analysis are indicated in Table S1.
(TIF)

Figure S2 Targeting strategy for the generation of the *Atxn2*-CAG42-knock-in model. Hatched rectangles represent *Atxn2* coding sequences, light grey symbolizes the CAG42-repeat and a solid line represents the chromosomal sequence. FRT sites are represented by double triangles and LoxP sites by single triangles. For primer sequences see Tables S2, S3, S4, S5.
(TIF)

Figure S3 Genotyping strategy. The genotyping strategy is based on the distinction between the homologous recombined Neo-excised knock-in (CAG42) allele (984 bp) and the wild-type (WT) allele (793 bp). Heterozygotes (CAG1/CAG42) showed both products.
(TIF)

Figure S4 Anti-ATXN2 immunohistochemistry of cerebellar Purkinje cells in 5 months old wild-type and Ataxin-2 deficient mice. Purkinje neurons of wild-type mice (WT) are selectively visualized by Ataxin-2 staining, while they do not display the specific immunoreactivity in Ataxin-2 deficient mice (KO).
(TIF)

Figure S5 Immunohistochemistry of cerebellar Purkinje cells in 24 months old wild-type and CAG42 mice. Calbindin immunoreactivity allowed the sensitive detection of Purkinje cell bodies and dendritic trees, but an alteration of signal intensity or cell numbers in mutant tissue was not detectable.
(TIF)

Figure S6 Overexpression of FBXW8 and CAG41-ATXN2 in HeLa cells. The soluble levels of Q22-ATXN2 and Q41-ATXN2 were unchanged after FBXW8 overexpression. For the insoluble levels, Q22-ATXN2 is again not influenced by FBXW8 levels, while a slight but insignificant reduction may be apparent for Q41-ATXN2, reminiscent of the stronger reduction for Q74-ATXN2 in Figure 8B. FBXW8 levels were increased by the overexpression both in the soluble and insoluble fraction (n = 3).
(TIF)

Table S1 Expected DNA fragment sizes of the designed Southern blot analysis. The digestion with NsiI allowed the verification of successful 5′ homologous recombination. SphI digestion specifically detected the genomic integration of the CAG repeat. The digestion with AvrII and SpeI allowed the detection of the 3′ homologous recombination.
(DOC)

Table S2 Primers for the verification of correct 3' and 5' integration.
(DOCX)

Table S3 Primers for generation of an internal 3' probe and external 5' probe for Southern Blot analysis.
(DOCX)

Table S4 Primers for the screening PCR to detect the Flp-mediated excision.
(DOCX)

Table S5 Expected fragment sizes for the Flp-excision Southern blot analysis. After Flp-excision, again the 3' probe was used to confirm the deletion.
(DOCX)
