# Supplementary figures and images for: ATXN2-CAG42 Sequesters PABPC1 into Insolubility and Induces FBXW8 in Cerebellum of Old Ataxic Knock-In Mice
Source: PLoS Genet. 2012 Aug 30;8(8):e1002920. doi: 10.1371/journal.pgen.1002920 (PMC3431311; doi:10.1371/journal.pgen.1002920)

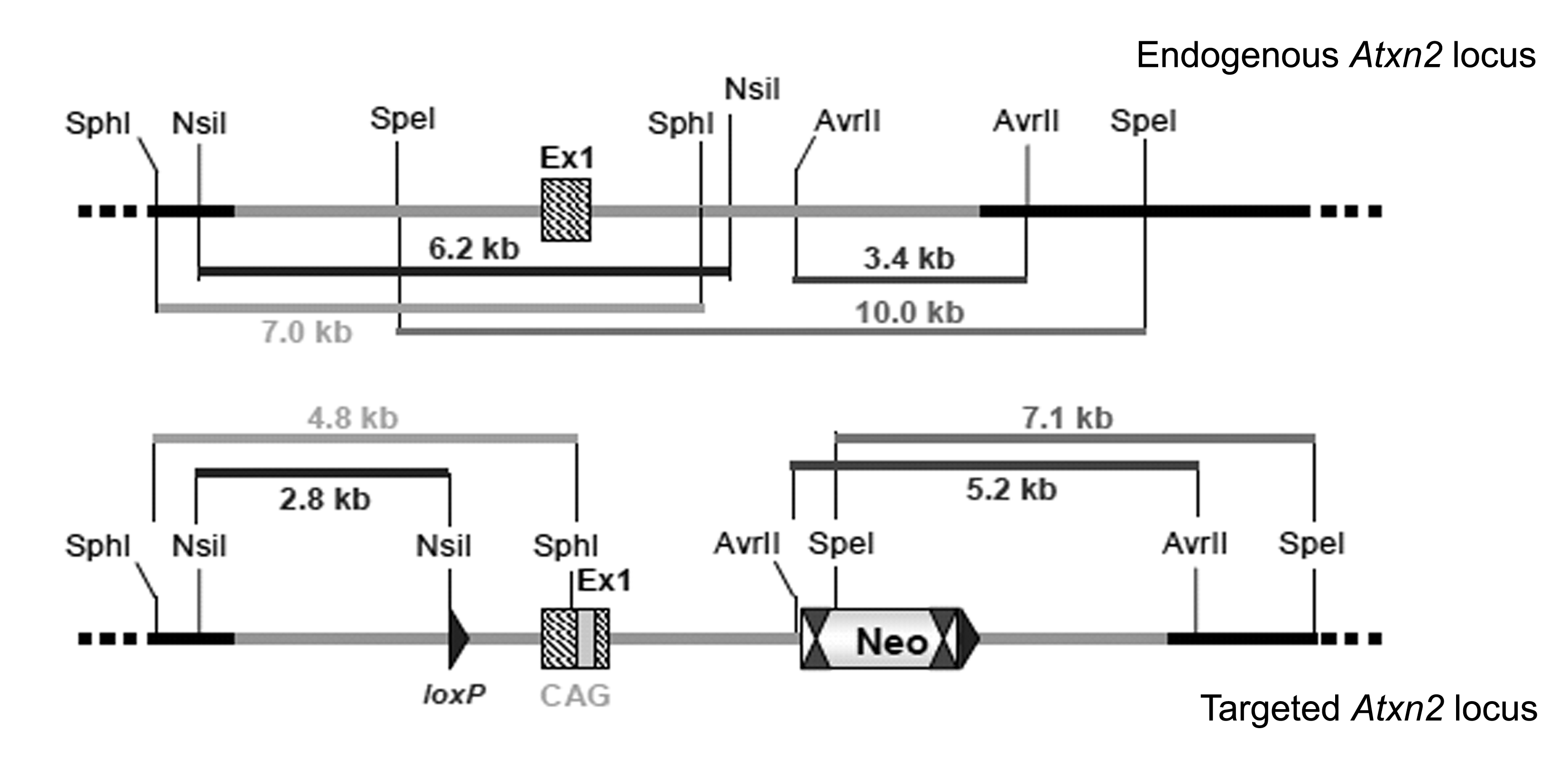

Supplement: Figure S1 — Southern blot strategy for the detection of homologous recombination at the Atxn2 locus. Exon 1 is symbolized by a hatched rectangle and the CAG repeat by a light grey rectangle. FRT sites are represented by grey double triangles and loxP sites by a single triangle. The expected DNA fragment sizes for the 5′ Southern analysis are indicated in Table S1. (TIF) [file pgen.1002920.s001.tif]

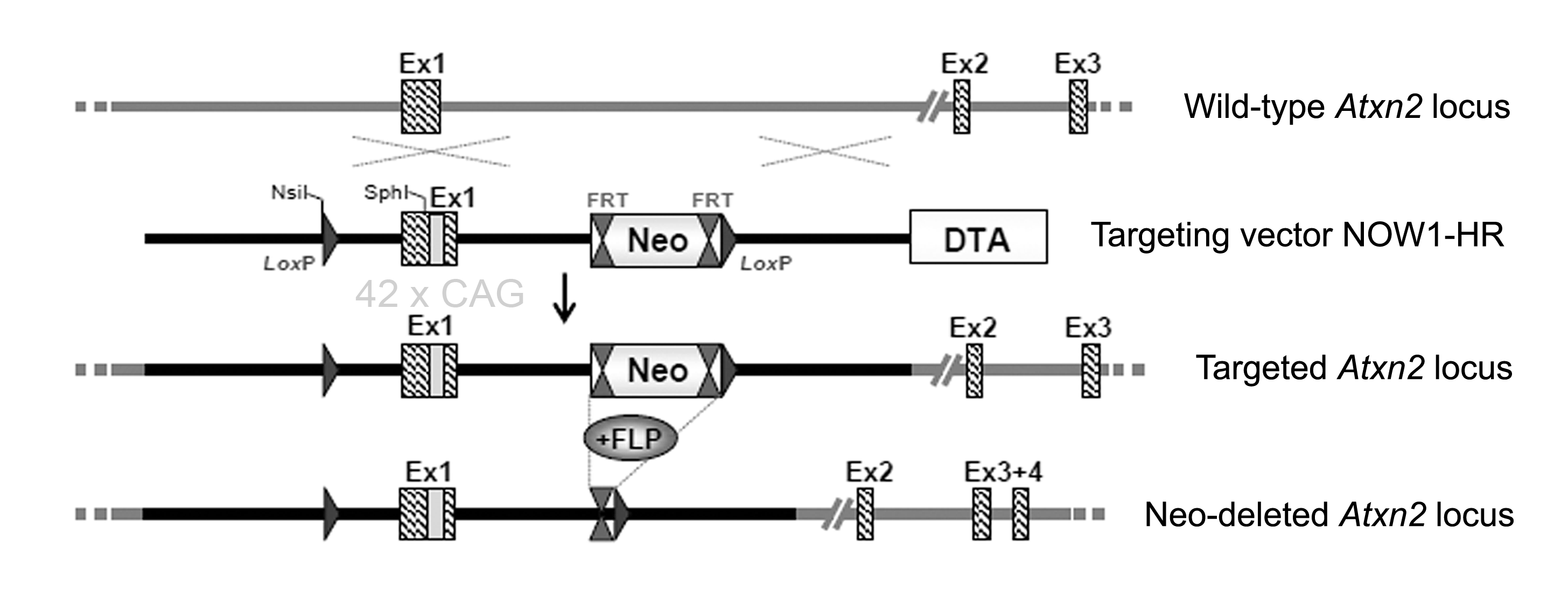

Supplement: Figure S2 — Targeting strategy for the generation of the Atxn2-CAG42-knock-in model. Hatched rectangles represent Atxn2 coding sequences, light grey symbolizes the CAG42-repeat and a solid line represents the chromosomal sequence. FRT sites are represented by double triangles and LoxP sites by single triangles. For primer sequences see Tables S2, S3, S4, S5. (TIF) [file pgen.1002920.s002.tif]

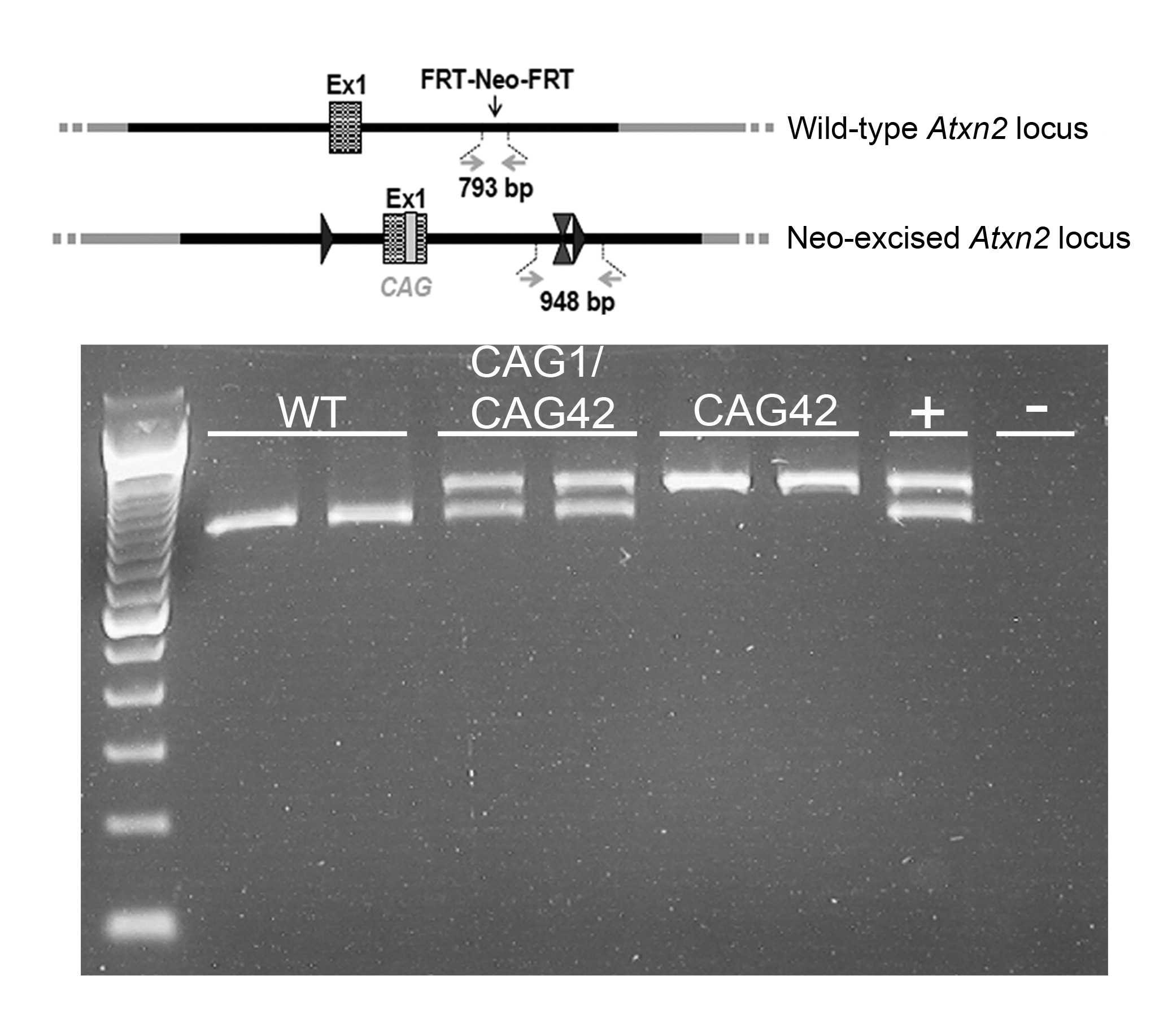

Supplement: Figure S3 — Genotyping strategy. The genotyping strategy is based on the distinction between the homologous recombined Neo-excised knock-in (CAG42) allele (984 bp) and the wild-type (WT) allele (793 bp). Heterozygotes (CAG1/CAG42) showed both products. (TIF) [file pgen.1002920.s003.tif]

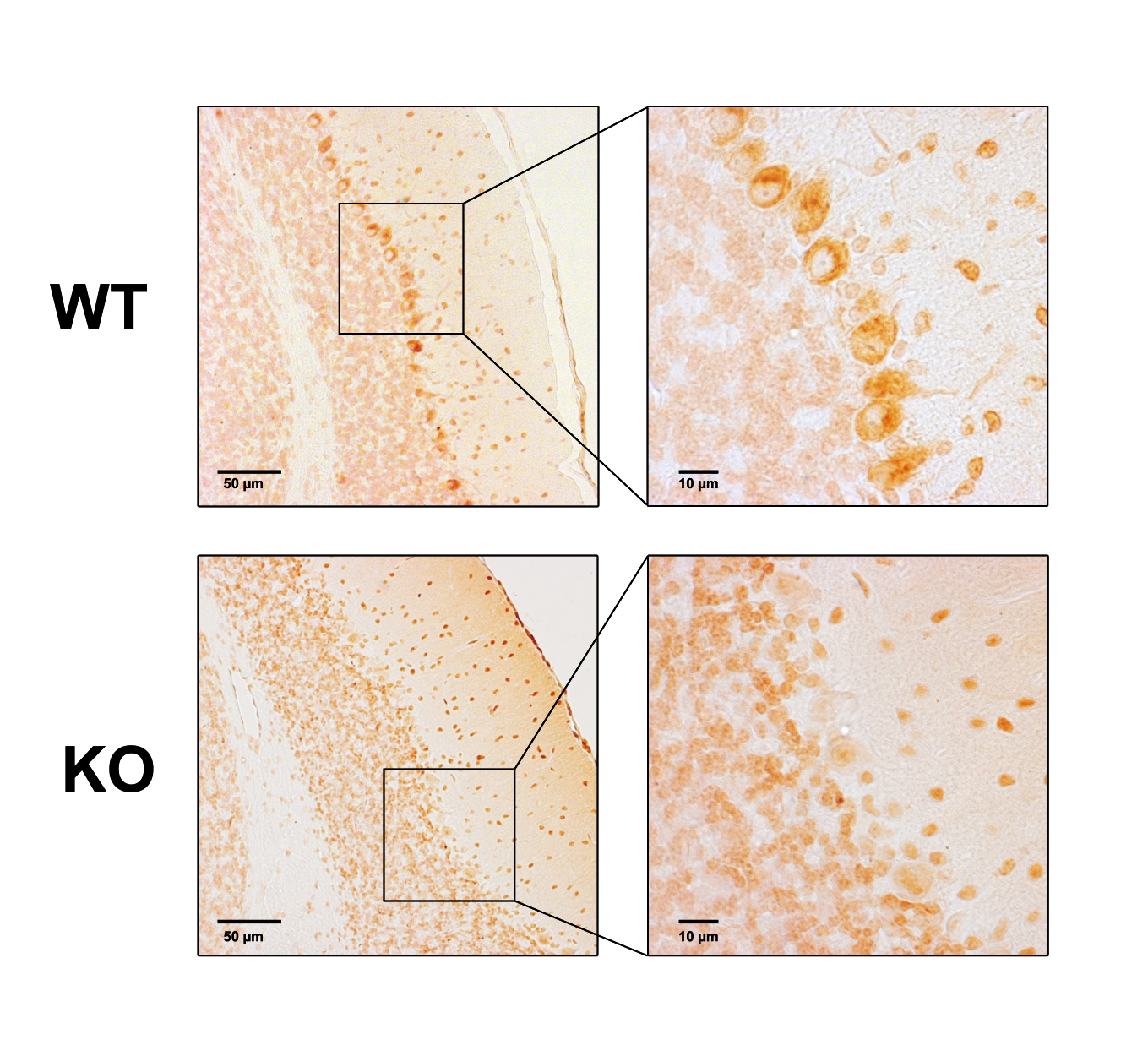

Supplement: Figure S4 — Anti-ATXN2 immunohistochemistry of cerebellar Purkinje cells in 5 months old wild-type and Ataxin-2 deficient mice. Purkinje neurons of wild-type mice (WT) are selectively visualized by Ataxin-2 staining, while they do not display the specific immunoreactivity in Ataxin-2 deficient mice (KO). (TIF) [file pgen.1002920.s004.tif]

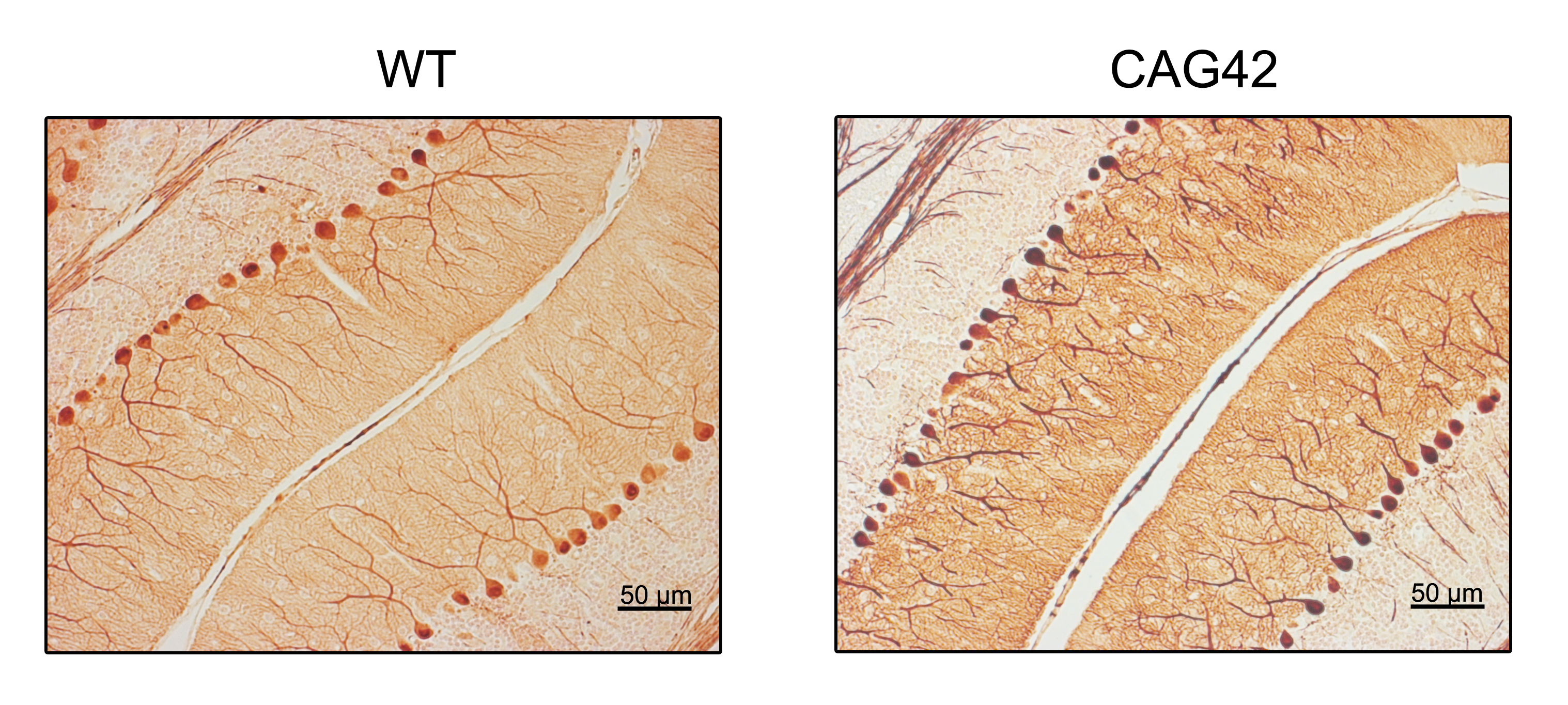

Supplement: Figure S5 — Immunohistochemistry of cerebellar Purkinje cells in 24 months old wild-type and CAG42 mice. Calbindin immunoreactivity allowed the sensitive detection of Purkinje cell bodies and dendritic trees, but an alteration of signal intensity or cell numbers in mutant tissue was not detectable. (TIF) [file pgen.1002920.s005.tif]

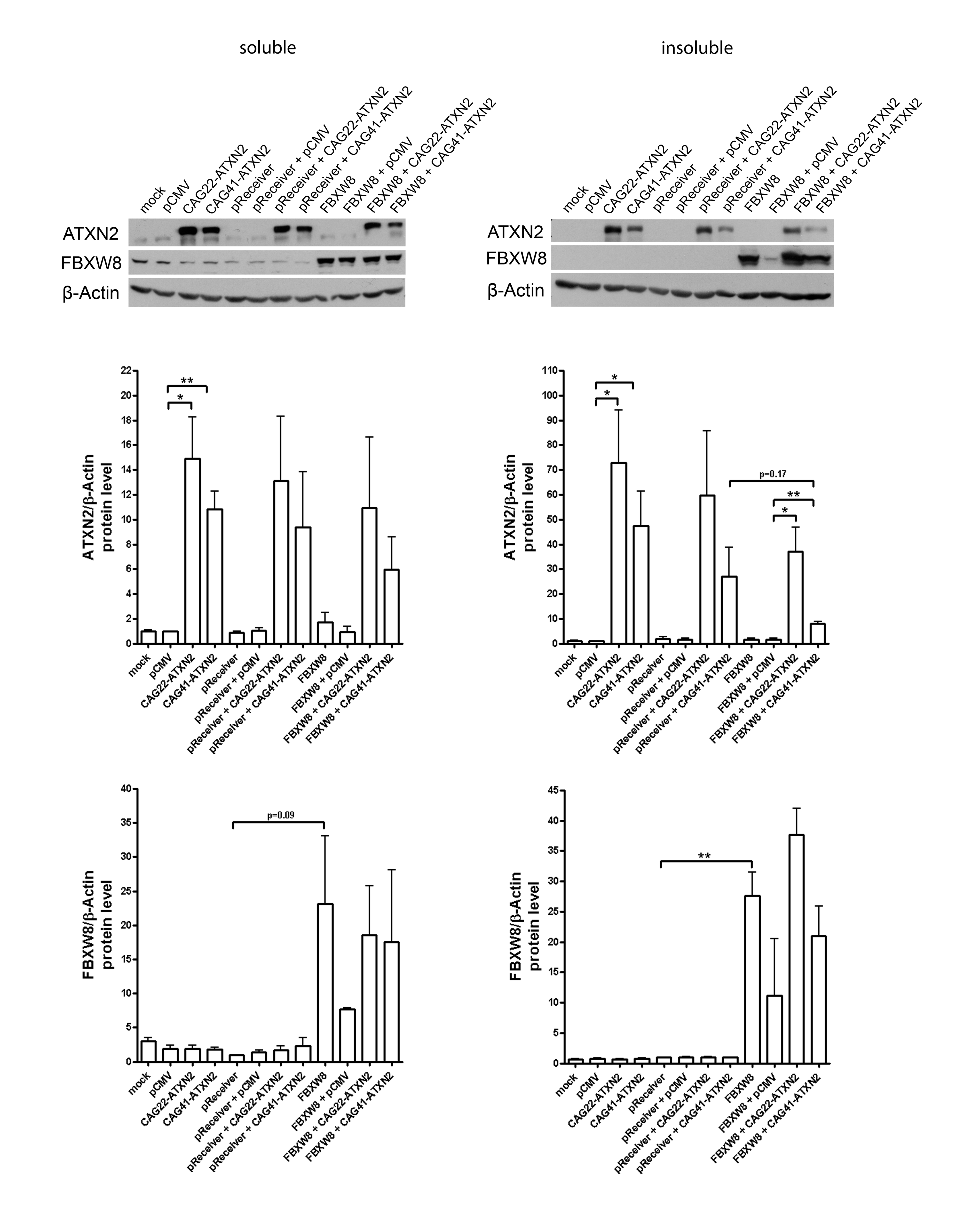

Supplement: Figure S6 — Overexpression of FBXW8 and CAG41-ATXN2 in HeLa cells. The soluble levels of Q22-ATXN2 and Q41-ATXN2 were unchanged after FBXW8 overexpression. For the insoluble levels, Q22-ATXN2 is again not influenced by FBXW8 levels, while a slight but insignificant reduction may be apparent for Q41-ATXN2, reminiscent of the stronger reduction for Q74-ATXN2 in Figure 8B. FBXW8 levels were increased by the overexpression both in the soluble and insoluble fraction (n = 3). (TIF) [file pgen.1002920.s006.tif]
