# Supplementary material for: ATXN2-CAG42 Sequesters PABPC1 into Insolubility and Induces FBXW8 in Cerebellum of Old Ataxic Knock-In Mice
Source: PLoS Genet. 2012 Aug 30;8(8):e1002920. doi: 10.1371/journal.pgen.1002920 (PMC3431311; doi:10.1371/journal.pgen.1002920)
Supplement: Table S1 — Expected DNA fragment sizes of the designed Southern blot analysis. The digestion with NsiI allowed the verification of successful 5′ homologous recombination. SphI digestion specifically detected the genomic integration of the CAG repeat. The digestion with AvrII and SpeI allowed the detection of the 3′ homologous recombination. (DOC) [file pgen.1002920.s007.doc]

Table S1

| **Genomic DNA digestion with enzyme** | **5’ homologous recombination** | | **3’ homologous recombination** | |
| --- | --- | --- | --- | --- |
|  | **exp. wild-type fragment size** | **exp. targeted fragment size** | **exp. wild-type fragment size** | **exp. targeted fragment size** |
| NsiI | 6.2 kb | 2.8 kb (*loxP* insertion) | / | / |
| SphI | 7.0 kb | 4.8 kb (CAG repeat insertion) | / | / |
| AvrII | / | / | 3.4 kb | 5.2 kb |
| SpeI | / | / | 10.0 kb | 7.1 kb |
