# Supplementary material for: ATXN2-CAG42 Sequesters PABPC1 into Insolubility and Induces FBXW8 in Cerebellum of Old Ataxic Knock-In Mice
Source: PLoS Genet. 2012 Aug 30;8(8):e1002920. doi: 10.1371/journal.pgen.1002920 (PMC3431311; doi:10.1371/journal.pgen.1002920)
Supplement: Table S2 — Primers for the verification of correct 3' and 5' integration. (DOCX) [file pgen.1002920.s008.docx]

Table S2. Primers for the verification of correct 3’ and 5’ integration

|  | **Primer** | **Sequence 5’-3’** | **PCR product size** |
| --- | --- | --- | --- |
| **3`-integration** | NOW1-A3 | CTATCAGGACATAGCGTTGGCTACC | 3132 bp |
|  | NOW1-B3 | CTGGTCTACAAAGTAGATGCTGCC |  |
| **5`-integration** | NOW1-C1 | TCCACTTTCCCAAACACAGGTCT | 2891 bp |
|  | NOW1-D1 | GGTCATCTTCACCTTCATATAACT | 3132 bp |
