# Supplementary material for: ATXN2-CAG42 Sequesters PABPC1 into Insolubility and Induces FBXW8 in Cerebellum of Old Ataxic Knock-In Mice
Source: PLoS Genet. 2012 Aug 30;8(8):e1002920. doi: 10.1371/journal.pgen.1002920 (PMC3431311; doi:10.1371/journal.pgen.1002920)
Supplement: Table S3 — Primers for generation of an internal 3' probe and external 5' probe for Southern Blot analysis. (DOCX) [file pgen.1002920.s009.docx]

Table S3. Primers for generation of an internal 3’ probe and external 5’ probe for Southern Blot analysis.

|  | **Primer** | **Sequence 5’-3’** | **Size of probe** |
| --- | --- | --- | --- |
| **3` probe** | NOW1-3I-D1 | CTTAACTTTGACCTTTTGTGAGGCTGTGC | 418 bp |
|  | NOW1-3I-D2 | TGAAAGCACATGCCTTGTTGAATGC |  |
| **5`probe** | NOW1-5E-G1 | TCCCAAACACAGGTCTGAGTAAGAAGC | 365 bp |
|  | NOW1-5E-G2 | CTGGGGAATCAAACTCAAAGTCTGC |  |
