# Supplementary material for: ATXN2-CAG42 Sequesters PABPC1 into Insolubility and Induces FBXW8 in Cerebellum of Old Ataxic Knock-In Mice
Source: PLoS Genet. 2012 Aug 30;8(8):e1002920. doi: 10.1371/journal.pgen.1002920 (PMC3431311; doi:10.1371/journal.pgen.1002920)
Supplement: Table S4 — Primers for the screening PCR to detect the Flp-mediated excision. (DOCX) [file pgen.1002920.s010.docx]

Table S4. Primers for the screening PCR to detect the Flp-mediated excision.

| **Primer** | **Sequence 5’-3’** | **PCR product in allele of** | | |
| --- | --- | --- | --- | --- |
| NOW1-K2 | TGAGTTGACTCCACAGGGAGGTGAGC | **WT** | **Targeted** | **Flp-excised** |
| NOW1-H2 | CCATCTCGCCAGCCCGTAAGATTC | 793 bp | 2944 bp | 948 bp |
