# Supplementary material for: ATXN2-CAG42 Sequesters PABPC1 into Insolubility and Induces FBXW8 in Cerebellum of Old Ataxic Knock-In Mice
Source: PLoS Genet. 2012 Aug 30;8(8):e1002920. doi: 10.1371/journal.pgen.1002920 (PMC3431311; doi:10.1371/journal.pgen.1002920)
Supplement: Table S5 — Expected fragment sizes for the Flp-excision Southern blot analysis. After Flp-excision, again the 3' probe was used to confirm the deletion. (DOCX) [file pgen.1002920.s011.docx]

**Table S5. Expected fragment sizes for the Flp-excision Southern blot analysis.
After Flp-excision, again the 3’ probe was used to confirm the deletion.**

| **Allele** | **Expected size of SpeI fragment** |
| --- | --- |
| wild-type | 10.1 kb |
| targeted | 7.2 kb |
| Flp-excised | 5.6 kb |
